# Improved myocardial mitochondrial energy metabolism in rats with chronic heart failure by modifying fatty acid oxidation using an extract of sand-fired aconite (Jianchang gang processing)

**DOI:** 10.3389/fphar.2025.1600410

**Published:** 2025-09-11

**Authors:** Hongtao Zhang, Yi Huang, Songhong Yang, Feipeng Gong, Yuncheng Gu, Qin Xie, Yanrong Ye, Xingmei Lu, Lingyun Zhong

**Affiliations:** ^1^ School of Pharmacy, Jiangxi University of Chinese Medicine, Nanchang, China; ^2^ Jiangxi Provincial People’s Hospital, The First Affiliated Hospital of Nanchang Medical College, Nanchang, China

**Keywords:** sand fired aconite slices, chronic heart failure, oxidative stress, fatty acid, energy metabolism, mitochondria

## Abstract

**Introduction:**

Sand-fired aconite slices (SFAS) demonstrate anti-heart failure effects, but the mechanism remains unclear. This study investigated myocardial mitochondrial energy metabolism as a therapeutic mechanism of SFAS in doxorubicin-induced chronic heart failure (CHF) rats.

**Methods:**

The CHF rat model was established via the intraperitoneal injection of doxorubicin (DOX). Following successful model production, rats were randomly assigned to nine groups. After drug administration, their cardiac function was assessed, and their cardiac tissue morphology and myocardial mitochondria were examined. Atrial natriuretic peptide (ANP), brain natriuretic peptide (BNP), norepinephrine (NE), malondialdehyde (MDA), superoxide dismutase (SOD), free fatty acid (FFA), sodium-potassium-ATPase (Na^+^-k^+^-ATPase), calcium-magnesium-ATPase (Ca^2+^-Mg^2+^-ATPase), and adenosine triphosphate (ATP) levels were quantified using enzyme-linked immunosorbent assays (ELISAs). Fatty acid translocase (CD36), carnitine palmitoyl transferase 1 (CPT1), adenosine 5′-monophosphate-activated protein kinase (AMPK), phosphorylated adenosine monophosphate-activated protein kinase (p-AMPK), peroxisome proliferator-activated receptor γ coactivator 1 alpha (PGC-1α), and Sirtuin 3 (SIRT3) protein expression levels were assessed by Western blot.

**Results:**

SFAS significantly improved cardiac function in CHF rats. It increased the left ventricular ejection fraction (LVEF) (from 34.22% ± 2.03%–83.68% ± 2.34%; *P* < 0.001) and left ventricular shortening fraction (LVFS) (from 17.06% ± 1.08%–53.86% ± 2.82%; *P* < 0.001) and decreased ANP (from 551.29 ± 14.63 pg/mL to 291.96 ± 11.28 pg/mL; *P* < 0.05), BNP (from 743.15 ± 18.03 pg/mL to 478.75 ± 10.57 pg/mL; *P* < 0.001), and NE levels (from 1,105.36 ± 21.79 pg/mL to 672.67 ± 6.70 pg/mL; *P* < 0.001). Additionally, it decreased MDA production (from 8.89 ± 0.36 nmol/mL to 5.11 ± 0.35 nmol/mL; *P* < 0.05) and increased SOD activity (from 264.82 ± 4.26 pg/mL to 529.64 ± 10.27 pg/mL; *P* < 0.001), Na^+^-K^+^-ATPase levels (from 7.19 ± 0.65 μmol/mL to 14.08 ± 0.28 μmol/mL; *P* < 0.001), Ca^2+^-Mg^2+^-ATPase levels (from 0.86 ± 0.03 μmol/mL to 1.40 ± 0.02 μmol/mL; *P* < 0.05), CD36 levels (*P* < 0.05), and CPT1 levels (*P* < 0.01). Moreover, it improved mitochondrial structural damage and reduced the level of oxidative stress in cardiomyocytes. Furthermore, SFAS promoted FFA oxidation (from 1,477.49 ± 7.60 μmol/mL to 768.87 ± 82.53 μmol/mL; *P* < 0.05) and ATP production (from 2,869.85 ± 298.26 nmol/mL to 5,483.17 ± 120.03 nmol/mL; *P* < 0.001) and increased p-AMPK, PGC-1α, and SIRT3 levels (*P* < 0.05 and *P* < 0.01).

**Conclusion:**

By activating the AMPK/PGC-1α/SIRT3 signaling pathway, SFAS ameliorated the impaired fatty acid oxidation pathway and enhanced mitochondrial function and antioxidant capacity in cardiomyocytes, ultimately reducing myocardial damage and restoring cardiac function in CHF rats.

## 1 Introduction

Chronic heart failure (CHF) is the terminal stage of cardiac disease, and it results from diverse etiologies. CHF results from pathological changes in the structure or function of the heart that lead to a decrease in cardiac output (Schwinger, 2021). Modern research indicates that the pathogenesis of CHF is closely linked to myocardial mitochondrial energy metabolism disorders (Wang et al., 2024). Although research on the pathology of CHF has deepened knowledge in recent years, progress in developing drugs to treat CHF remains relatively slow, and the prognosis of patients with CHF remains poor due to its complex etiology. Therefore, discovering new drugs to better treat CHF is required to improve the survival rate and prognosis of patients with all types of CHF.

Often termed the body’s “engine,” the heart exhibits the most vigorous energy metabolism among human organs and is primarily composed of myocardial tissue. As the main site of myocardial energy metabolism, mitochondria supply energy to the heart through fatty acid oxidation, which is particularly important for the maintenance of the normal physiological function of the heart (Lopaschuk et al., 2021). Disruption of cardiomyocyte mitochondrial energy metabolism is a key contributor to CHF. When the structure of myocardial mitochondria changes, oxidative stress increases, mitochondrial damage intensifies, energy metabolic pathways, such as fatty acid β oxidation, are affected and transformed, and defective myocardial energy metabolism enters a vicious cycle, ultimately leading to CHF (Li et al., 2023). Therefore, using cardiac energy metabolism as the starting point and promoting fatty acid oxidation is an effective way to treat CHF.

Traditional Chinese medicine (TCM) boasts a long history of application and rich clinical experience in treating CHF. Characterized by multi-target effects and relatively fewer side effects, TCM represents a valuable therapeutic option (Wang et al., 2022; Zeng et al., 2023; Qi et al., 2024; Li et al., 2024). Within TCM theory, CHF manifestations are often classified under syndromes such as “palpitations.” TCM theory postulates that CHF is mostly due to prolonged illness and physical weakness (Gao et al., 2023). Modern pharmacological studies confirm that *Aconitum carmichaelii* Debx (Ranunculaceae; Aconiti Lateralis Radix Praeparata) possesses anti-heart failure effects, with its mechanism linked to the improvement of cardiac mitochondrial energy metabolism (Xu et al., 2021). However, due to the potent and rapid-acting nature of raw aconite, processing it to reduce toxicity is essential, and preparations made using different methods exhibit distinct therapeutic profiles. Jianchang gang is a renowned TCM processing school originating in Southern China. It inherits the sand-fired aconite slices (SFAS) and dried ginger steamed aconite slices (DGSAS) processing methods. Among these, SFAS, prepared by stir-frying sliced aconite with heated sand, has demonstrated efficacy in treating CHF in clinical and preclinical settings; however, the precise mechanism underlying SFAS’s anti-CHF effects remains incompletely elucidated. Particularly, its impact on myocardial energy metabolism has not been clarified. Therefore, this study used the fatty acid oxidation pathway of myocardial mitochondrial energy metabolism as the entry point to explore the effects of SFAS on the cardiac function of doxorubicin (DOX)-induced CHF rats and the related mechanisms of action, providing a reliable reference for the discovery of drugs for treating CHF and the study of its pharmacological mechanisms of action.

## 2 Materials

### 2.1 Plant material and authentication

Salted aconite roots from Jiangyou, Sichuan, China, were purchased from Jiangxi Guhan Refined Chinese Herbal Pieces Co., Ltd. (Nanchang, lot number 20231001). The botanical identity of the source material, *Aconitum carmichaelii* Debx. (Ranunculaceae; Aconiti Lateralis Radix Praeparata cum Salo), was confirmed by Professor Fei Ge (Jiangxi University of Chinese Medicine). Additionally, adjuvants used in processing the plant included *Glycyrrhiza uralensis* Fisch. (Fabaceae; Glycyrrhizae Radix et Rhizoma), *Zingiber officinale* Rosc. (Zingiberaceae; Zingiberis Rhizoma Recens), and *Glycine max* (L.) Merr. (Fabaceae; Glycines Semen Nigrum). Professor Fei Ge confirmed the botanical identities of these plants. Voucher specimens of the raw material, adjuvants, and representative samples of the final processed products have been deposited in the medicinal herb storeroom of the Jiangxi University of Chinese Medicine, Nanchang, China.

### 2.2 Animal

One hundred and thirty-two specific pathogen-free male Sprague–Dawley rats (SD rats; 180–200 g) were purchased from SPF Biotechnology Co., Ltd. (experimental animal production license No. SCXK 2019–0010, Beijing; experimental unit use license No. SYXK 2022–0002, Gan). The Ethics Committee of Jiangxi Provincial People’s Hospital approved the study. Rats were housed under standard conditions (temperature: 22 °C–25 °C; humidity: 50% ± 5%; 12-h light/dark cycle) with *ad libitum* access to standard rodent chow and water, in compliance with relevant guidelines for experimental animal care. The Ethics Committee of Jiangxi Provincial People’s Hospital” to align with the expression in the following text (the two have the same meaning, both referring to “the ethics committee of Jiangxi Provincial People’s Hospital”). approved all animal experiments (No. KT-071), and animal experiments and related procedures were performed in accordance with the institution’s guidelines for animal experiments.

### 2.3 Reagents and instruments

Doxorubicin hydrochloride was purchased from Shanghai Maclin Biochemical Technology Co., Ltd. (Batch No. C15533026). Captopril was purchased from Guangdong Bidi Pharmaceutical Co., Ltd. (Batch No. 20230602A). Isoflurane was purchased from Shanghai Aladdin Biochemical Technology Co., Ltd. (Batch No. Lot#K2123447). Masson Tri-color dyeing kit was purchased from Beijing Solaibao Technology Co., Ltd. (Batch No. G1340). 5′AMP-activated protein kinase (AMPK) antibodies (Batch No. ab32047), fatty acid translocase (CD36) antibodies (Batch No. ab252923), and phosphorylated AMP-activated protein kinase (p-AMPK; Batch No. ab133448) were purchased from Abcam Company. Sirtuin 3 (SIRT3) antibodies (Batch No. 10099-1-AP), peroxisome proliferator-activated receptor coactivator-1α (PGC-1α) antibodies (Batch No. 66369-1-Ig), and carnitine palmitoyl transferase 1 (CPT1) antibodies (Batch No. 15184-1-AP) were purchased from Wuhan Sanying Biotechnology Co., Ltd. Atrial natriuretic peptide (ANP) Kits (Batch No. MK6949A), brain natriuretic peptide (BNP; Batch No. MK1608A), norepinephrine (NE; Batch No. MK2097A), malondialdehyde (MDA; Batch No. MK6877A), superoxide dismutase (SOD; Batch No. MK1927A), sodium-potassium-ATPase (Na^+^-k^+^-ATPase; Batch No. MK6748A), calcium-magnesium-ATPase (Ca^2+^-Mg^2+^-ATPase; Batch No. MK0007RA), adenosine triphosphate (ATP; Batch No. MK6913A), and free fatty acid (FFA; Batch No. MK1865A) kits were purchased from Jiangsu Sumeike Biotechnology Co., Ltd.

We used a Fresco17 refrigerated centrifuge (Thermo Fisher company), Vevo2100 ultra-high resolution small animal color doppler ultrasound real-time imaging system (Visual Sonics company), Paraffin embedding machine, EG1150 (Leica company), Paraffin slicer RM2016 (Leica company), MF43 Microscope (Mingmei photoelectric technology Co., Ltd.), MC50 digital imaging measurement and analysis system (Mingmei photoelectric technology Co., Ltd.), Infinite F50 automatic enzyme label analyzer (Tecan company), Ultra-low temperature refrigerator (Haier company), and JEM-1400 transmission electron microscope (JEOL company) for the study.

## 3 Methods

### 3.1 Sample preparation

#### 3.1.1 Medicinal material processing

**FIGURE 1:**
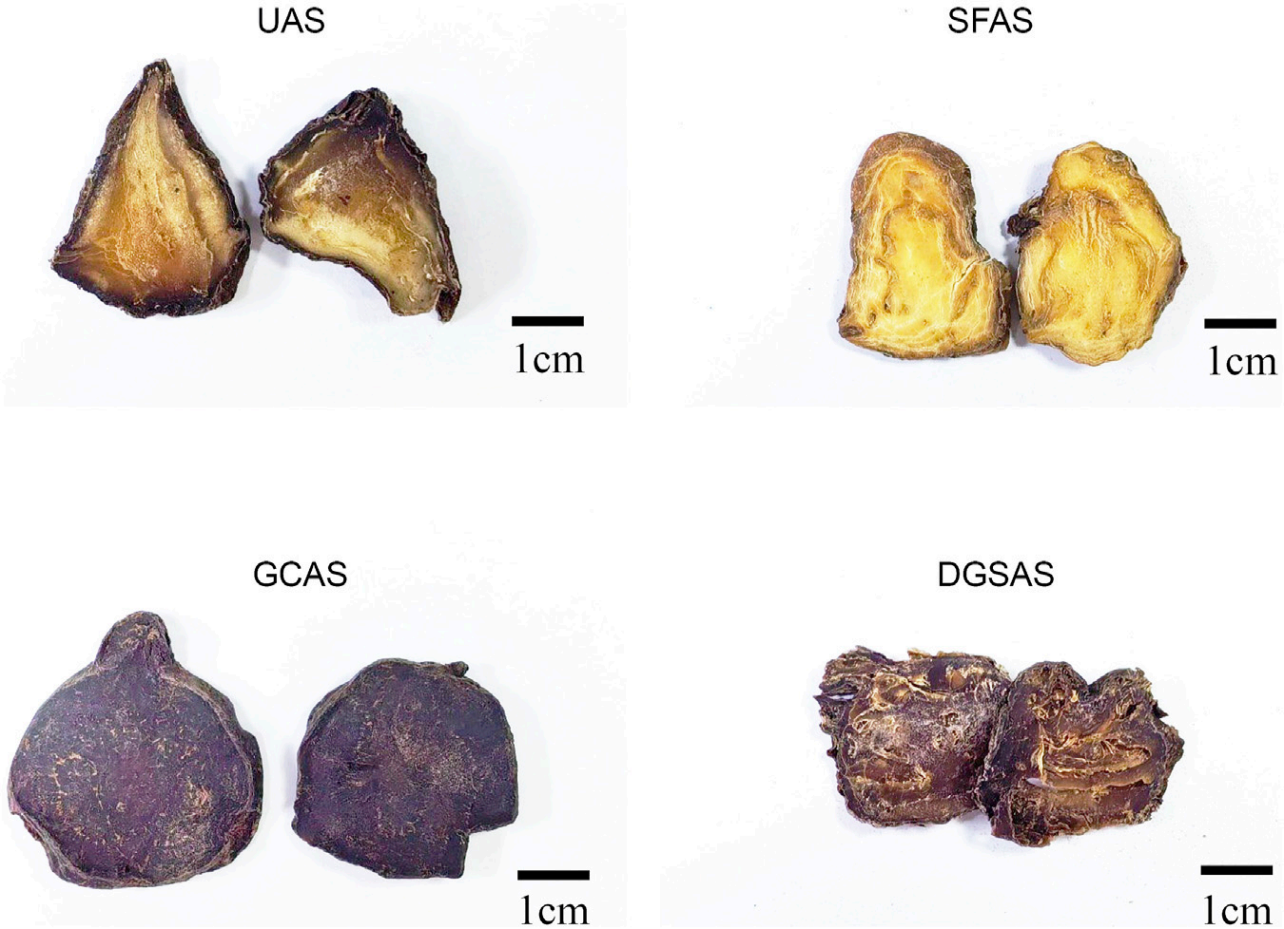
Sample images.

Unprocessed aconite slices (UAS): The salted aconite was cleaned by stirring in water, soaked until the saltiness reduces, thinly sliced, and sun-dried. (Figure 1).

Sand-fired aconite slices (SFAS): The salted aconite was cleaned by stirring in water. Its skin was then scraped off, and it was cut into thick slices. These slices were soaked until the saltiness reduces, after which it was removed and dried. Clean sand was fried in a wok until smooth. Botanical drugs were added to the sand and stirred until their sections bulk up and turn white and yellow. Afterwards, the botanical drugs were removed, the river sand was sieved, and the drugs were left to dry (Figure 1).

Glycyrrhiza cooked aconite slices (GCAS): The salted aconite was cleaned by stirring in water, soaked until saltiness reduces, and boiled with licorice and black beans until the mouth is cut and tongue does not numb. Afterwards, the adjuvant herbs were removed, and the aconite was thinly sliced and dried (Figure 1).

Dried ginger steamed aconite slices (DGSAS): The salted aconite was cleaned by stirring in water, soaked until saltiness reduces, and dried. It was then moistened with ginger juice, steamed, removed, dried until 70% dehydrated, sliced, and dried (Figure 1).

All processed products (UAS, SFAS, GCAS, and DGSAS) were prepared according to optimized procedures meeting established quality standards.

#### 3.1.2 Ultra-performance liquid chromatography-tandem mass spectrometry (UPLC-MS/MS)

Chemical profiling and quality control were performed using ultra-performance liquid chromatography-tandem mass spectrometry (UPLC-MS/MS; Agilent 1,260 ultra-high performance liquid chromatography system, Agilent, the United States of America; AB TRIPLE QUDA 4,500 mass spectrometer, AB SCIEX, the United States of America).

**FIGURE 2:**
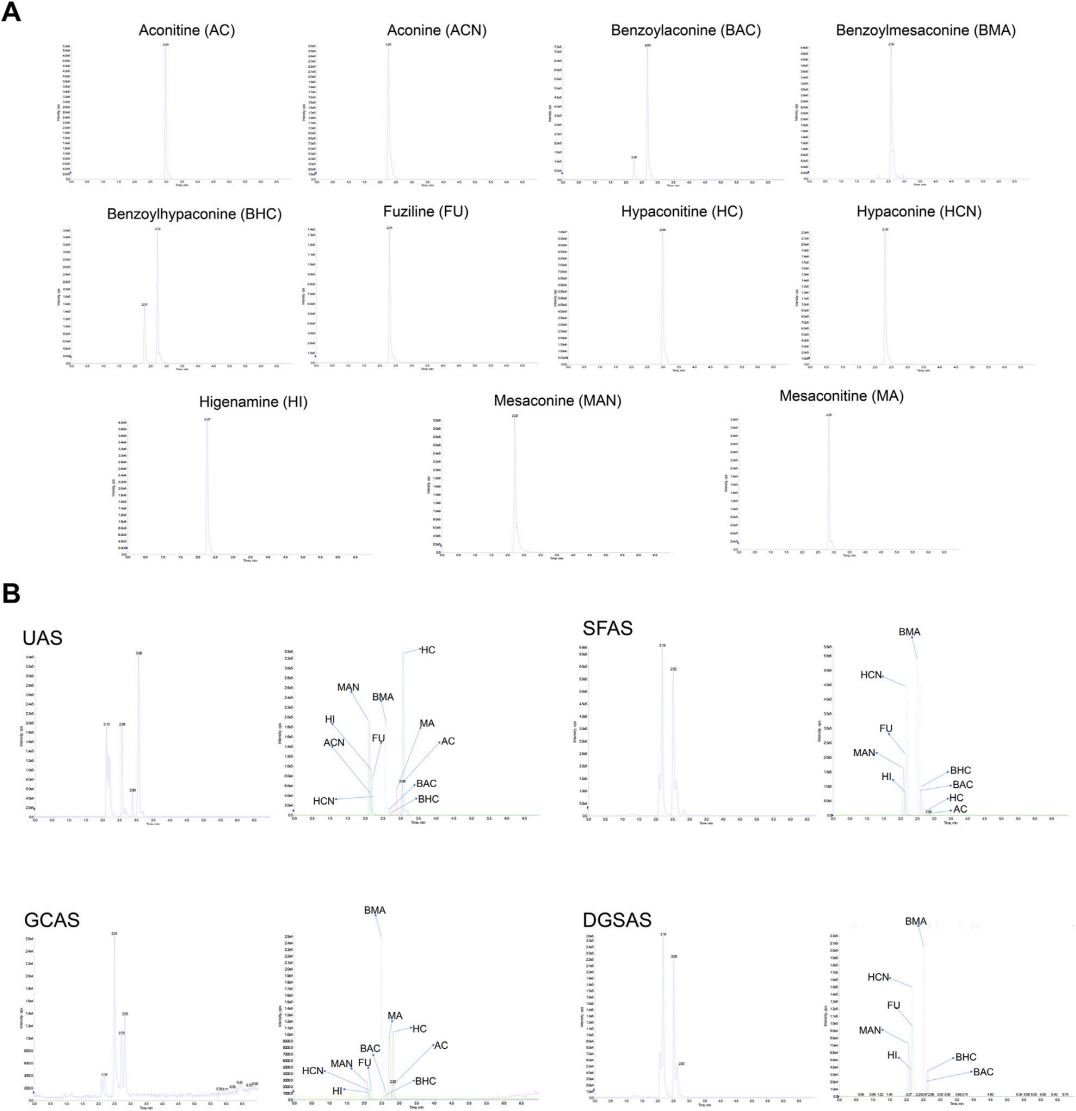
Chemical composition testing. **(A)** Standard materials of each component. **(B)** Components of each sample.

As shown in Figure 2, the extract ion chromatography (XIC) chromatogram of UAS and GCAS showed 11 characteristic peaks, including Aconitine (AC), Mesaconit (MA), Benzoylaconine (BAC), Benzoylmesaconine (BMA), Aconine (ACN), Mesine (MAN), Hypaconitine (HC), Benzoylhypaconine (BHC), Hypaconine (HCN), Fuziline (FU), and Higenamine (HI). The XIC chromatogram of SFAS had nine characteristic peaks, including AC, HC, HI, BAC, BHC, BMA, HCN, FU, and MAN, and that of DGSAS had eight characteristic peaks, including AC, HI, MAN, FU, HCN, BMA, BHC, and BAC. SFAS differed from UAS, GCAS, and DGSAS in the main toxic components, including AC and HC, and low-toxic high-efficiency cardiotonic components, including BMA and HCN, which are closely associated with the toxicity profile and therapeutic efficacy of each preparation (Figure 2).

#### 3.1.3 Decoction preparation

Processed materials (10× water) were soaked (45 min), boiled (30 min), and re-extracted (6× water, 30 min). Combined decoctions were concentrated to 0.15 g/mL under reduced pressure and stored at 4 °C.

### 3.2 Animal modeling and experimental design

#### 3.2.1 CHF induction and group assignment

Male SD rats (n = 132) were randomized into Control (n = 12; saline) and CHF model (n = 120; doxorubicin 18 mg/kg ip. delivered via eight injections over 4 weeks) groups.

Successful CHF induction was confirmed by echocardiography (LVEF <40% and LVFS <30%). Survivors (n = 90) were stratified by weight and randomly allocated to 9 groups (n = 10/group):

Based on the weight range, they were divided into four strata (Weight_min_ + n × 30 g). Rats within each stratum were then randomly allocated to experimental groups using a random number table. The final experimental groups (n = 10 per group) were the CHF model, captopril, unprocessed aconite slices-high dose (UAS-H), unprocessed aconite slices-low dose (UAS-L), sand-fired aconite slices high-dose (SFAS-H), sand-fired aconite slices-low dose (SFAS-L), GCAS, and DGSAS groups.

#### 3.2.2 Drug and dosage instructions

Control and CHF model groups: administered normal saline.

Captopril group: positive control (administered 2.63 mg/kg of captopril; clinical dose-derived: 12.5 mg per dose, twice daily).

UAS: SFAS precursor (high dose group: administered 1.58 g/kg of UAS; low dose group: administered half the dose received by the high dose group).

SFAS: test compound (high dose group: administered 1.58 g/kg of SFAS; low dose group: administered half the dose received by the high dose group).

GCAS: standard processed aconite (administered 1.58 g/kg of GCAS).

DGSAS: Jianchang gang processed aconite (administered 1.58 g/kg of DGSAS).

The high dose (1.58 g/kg) was derived from the maximum clinical aconite dose (15 g, Chinese Pharmacopoeia 2020); the low dose (0.8 g/kg) was half the high dose.

#### 3.2.3 Treatment protocol

**FIGURE 3:**
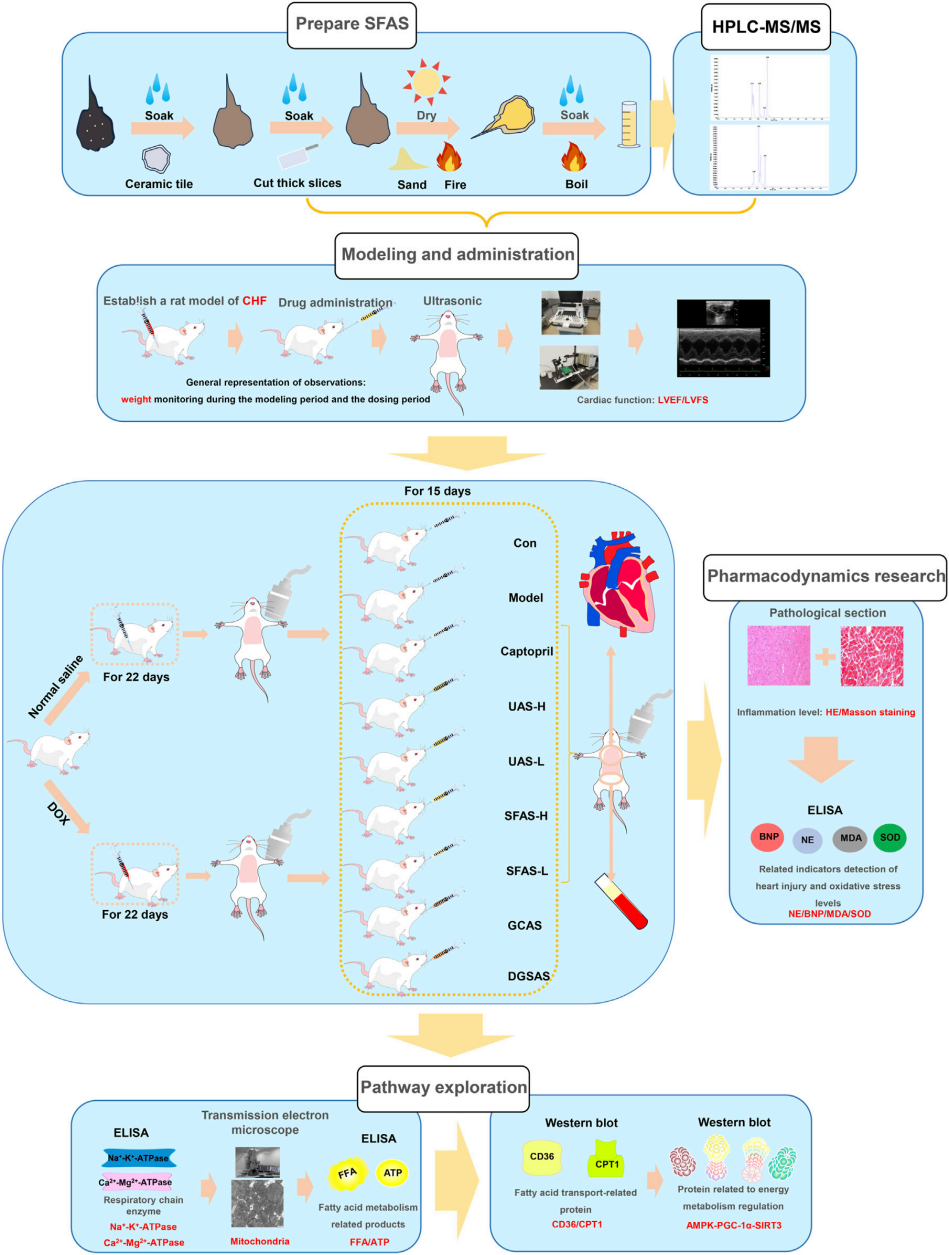
Technical roadmap.

Treatments were administered daily via oral gavage for 15 days. Cardiac function was reassessed post-treatment (Figure 3).

### 3.3 Sample collection and processing

After the final echocardiography, fasted rats were anesthetized by administering sodium pentobarbital 50 mg/kg ip. Blood was collected from the abdominal aorta, centrifuged (3,000 rpm, 20 min, 4 °C), and serum was stored at −80 °C. Cardiac tissues were sectioned and processed as follows:

Histopathology: 4% paraformaldehyde fixation.

Molecular analysis: Snap-frozen in liquid nitrogen (−80 °C storage).

### 3.4 Physiological monitoring

Body weight, behavioral responses, fur condition, and feeding patterns were recorded throughout the study.

### 3.5 Indicator detection

#### 3.5.1 Echocardiography

Under isoflurane anesthesia (3% induction, 1%–2% maintenance), cardiac parameters (LVEF and LVFS) were measured using a Vevo2100 system, with at least three cardiac cycles analyzed per rat.

#### 3.5.2 Histopathological analysis

##### 3.5.2.1 Hematoxylin and eosin (H&E) staining

Heart tissues were fixed in 4% paraformaldehyde (>48 h), paraffin-embedded, sectioned, baked, dewaxed in xylene, rehydrated, and subjected to HE staining. After dehydration, clearing, and mounting, cardiac cell morphology was assessed per group using light microscopy.

##### 3.5.2.2 Masson’s trichrome (masson) staining

Heart tissue was fixed in 4% paraformaldehyde for 48 h, paraffin-embedded, and sectioned. Following baking at 60 °C for 2 h and standard dewaxing/rehydration, sections were stained, dehydrated, cleared in xylene, and mounted with neutral gum. Cardiac collagen and muscle fibers were then visualized using light microscopy. ImageJ software was used for semi-quantitative analysis of the images.

#### 3.5.3 Enzyme-linked immunosorbent assay

Serum NE and myocardial tissue biomarkers (ANP, BNP, MDA, SOD, Na^+^-K^+^-ATPase, Ca^2+^-Mg^2+^-ATPase, ATP, and FFA) were quantified using commercial ELISA kits according to manufacturer protocols.

#### 3.5.4 Western blot

Myocardial tissue samples frozen at −80 °C were thawed at 4 °C. AMPK, PGC-1α, SIRT3, CD36, and CPT1 expression levels in rat myocardial tissue were determined by Western blot.

Myocardial tissues stored at −80 °C were thawed at 4 °C. Total protein was extracted using a radio-immunoprecipitation assay (RIPA) lysis buffer, and concentrations of proteins were quantified via a bicinchoninic acid (BCA) assay. Proteins (AMPK, PGC-1α, SIRT3, CD36, and CPT1) were separated by sodium dodecyl sulfate (SDS)-polyacrylamide gel electrophoresis (PAGE; 10% separating gel, 4% stacking gel) under constant voltage (80V stacking gel, 120V separating gel) with cooling. Following electrophoresis, proteins were transferred to nitrocellulose membranes (300 mA constant current; transfer duration optimized to the molecular weight of each protein).

Membranes were blocked and incubated overnight at 4 °C with primary antibodies (e.g., AMPK or SIRT3 at 1:2,000 dilution), followed by incubation with HRP-conjugated secondary antibodies (1:5,000, 1 h 10 min, RT). After tris-buffered saline with Tween 20 (TBST) washes, protein bands were detected using enhanced chemiluminescence (ECL) under auto-exposure.

### 3.6 Transmission electron microscopy (TEM)

Cardiac tissue was fixed in 2.5% glutaraldehyde, washed with PBS (phosphate-buffered saline), and post-fixed in 1% osmium tetroxide for 2 h. After PBS washes, samples were dehydrated at 4 °C, resin-embedded at room temperature, and polymerized. Ultrathin sections (70–90 nm) were post-stained with uranyl acetate and lead citrate, then examined by TEM.

### 3.7 Statistical analysis

SPSS 26.0 software was used for statistical analysis, and Graphpad Prism 9.5 software was used for plotting. Statistical differences among multiple groups were assessed using one-way ANOVA (for data meeting assumptions of normality and homogeneity of variance, including body weight, LVEF, LVFS, collagen deposition, BNP, SOD, Na^+^-K^+^-ATPase, ATP, NE, p-AMPK, CD36, CPT1, PGC-1α, and SIRT3). Kruskal-Wallis test was performed for non-normally distributed data (ANP, MDA, and Ca^2+^-Mg^2+^-ATPase), and Welch’s ANOVA was performed for normally distributed data with unequal variances (FFA and AMPK). A *P* value <0.05 was considered statistically significant.

## 4 Result

### 4.1 Changes in general physical signs of rats

**FIGURE 4:**
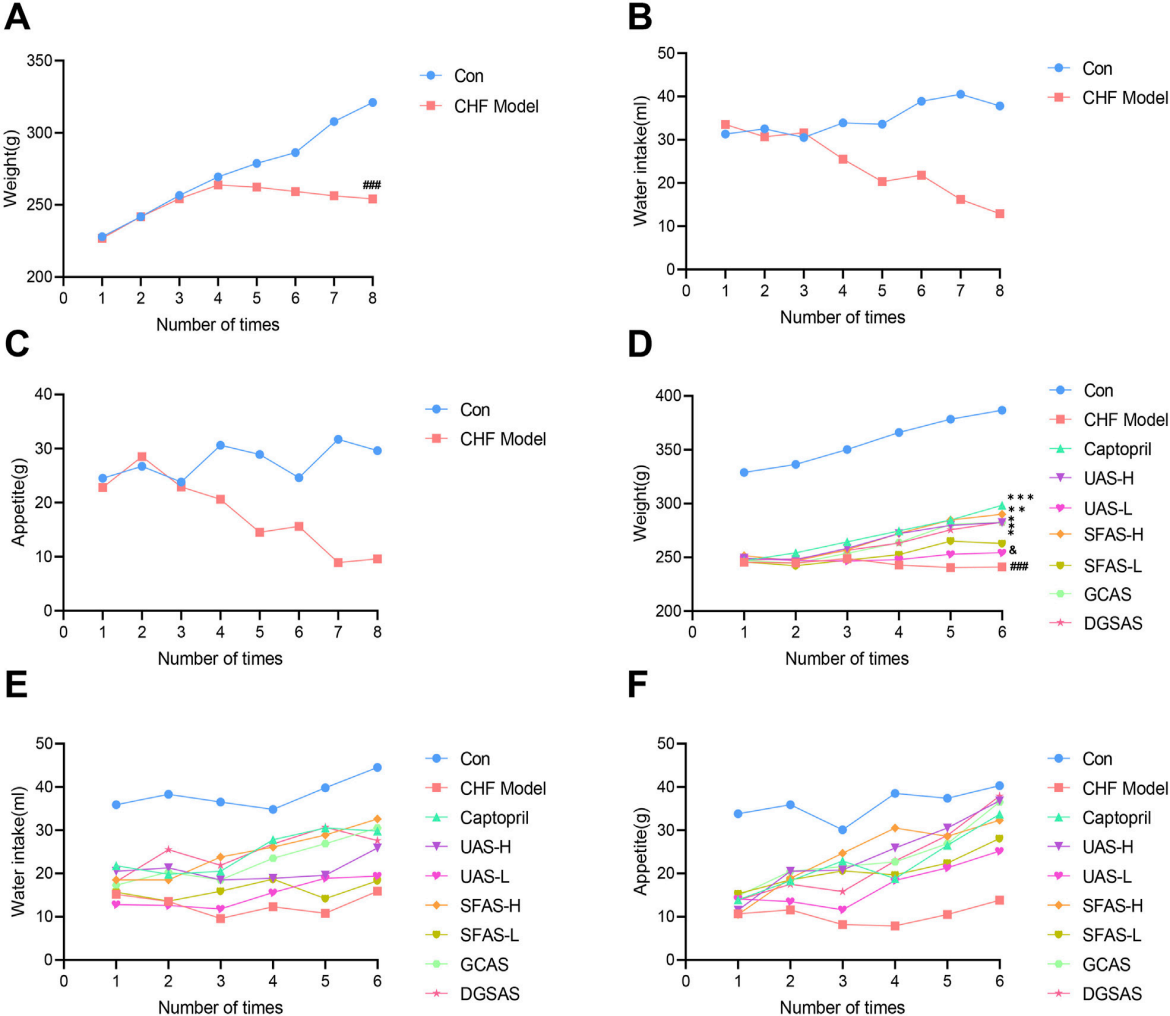
The entire experiment and changes in vital signs. **(A)** Changes in the body weight of rats during CHF rat model development. **(B)** Changes in water intake by rats during CHF rat model development. **(C)** Changes in the appetite of rats during CHF rat model development. **(D)** Changes in the body weight of rats in each group during drug administration. **(E)** Changes in water intake by rats in each group during drug administration. **(F)** Changes in the appetite of rats in each group during drug administration. ^###^ *P* < 0.001 in comparisons with the control group; ^*^ *P* < 0.05, ^**^ *P* < 0.01, and ^***^ *P* < 0.001 in comparisons with the CHF model group; ^&^ *P* < 0.05 in comparisons with the SFAS-H group.

During the CHF rat model development, control rats exhibited normal growth, with normal drinking and eating, soft and shiny fur, quick movements, large size, and gradually increasing weight. Conversely, rats in the CHF model group displayed significantly stunted growth or even weight loss, with a significant decrease in water and food intake, rough, easily shed fur, slow reactions, reduced activity, increased huddling behavior, and significantly lower weight than the control group (*P* < 0.001; Figure 4A). Some rats had severe ascites, and when the cumulative dose of DOX reached 18 mg/kg, the rats showed obvious signs of heart failure (Figures 4A–C).

During the drug administration phase of the study, the rats in the CHF model group still showed a dull response, dull fur, a downward trend in weight, and lower weights than those of the control group *(P* < 0.001; Figure 4D), and the signs of heart failure became more obvious. Compared with the CHF model group, rats in each drug administration group showed improvement in the above aspects (Figures 4E,F). Apart from rats in the UAS-L and the SFAS-L groups, rats in the captopril, SFAS-H, GCAS, and DGSAS groups had significantly higher body weights than those of the rats in the CHF model group (*P* < 0.05, *P* < 0.01, or *P* < 0.001; Figure 4D), and the signs of heart failure improved. Notably, although the weight of the rats in the UAS-H group, the UAS-L group, and the SFAS-L group increased during the drug administration period, some rats still showed obvious ascites, and mortalities occurred. Notably, the UAS-L group exhibited lower body weight than the SFAS-H group.

### 4.2 SFAS improved cardiac function in CHF rats

**FIGURE 5:**
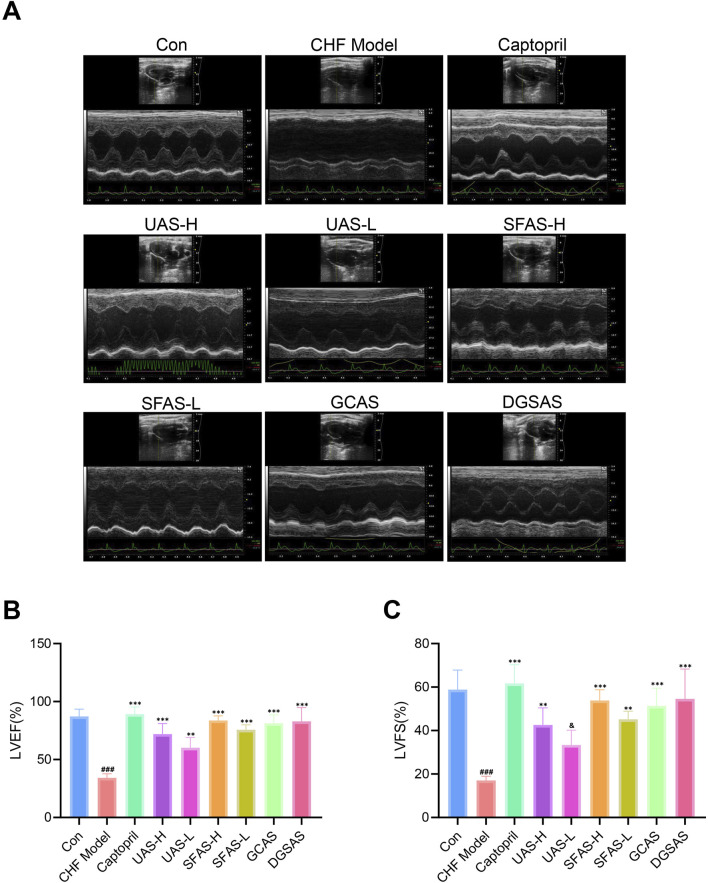
Ultrasound scan of rats in each group. **(A)** Ultrasound atlas of rats in each group (*n* = 3). **(B)** LVEF of rats in each group (*n* = 3). **(C)** LVFS of rats in each group (*n* = 3). ^###^ *P* < 0.001 in comparisons with the control group; ^**^ *P* < 0.01 and ^***^ *P* < 0.001 in comparisons with the CHF model group; ^&^ *P* < 0.05 in comparisons with the SFAS-H group.

The cardiac ultrasound results showed that LVEF and LVFS in the CHF model group were significantly decreased compared to those in the control group (*P* < 0.001 Figures 5B,C), indicating impaired systolic function and significantly reduced cardiac output in CHF model rats. Compared with the CHF model group, the LVEF of rats in all drug administration groups was significantly higher (*P* < 0.001; Figure 5B). Additionally, LVFS significantly improved in all treatment groups except the UAS-L group (*P* < 0.01 or *P* < 0.001; Figure 5C), indicating that captopril and the various products can significantly improve the cardiac function of CHF rats. Among them, SFAS-H and captopril had similar curative effects (*P* > 0.05), and the improvement in cardiac function in the SFAS-H group was significantly better than that in the SFAS-L group (*P* < 0.05; Figures 5A–C).

### 4.3 SFAS improved the pathology of heart tissue in CHF rats

#### 4.3.1 Hematoxylin and eosin staining

**FIGURE 6:**
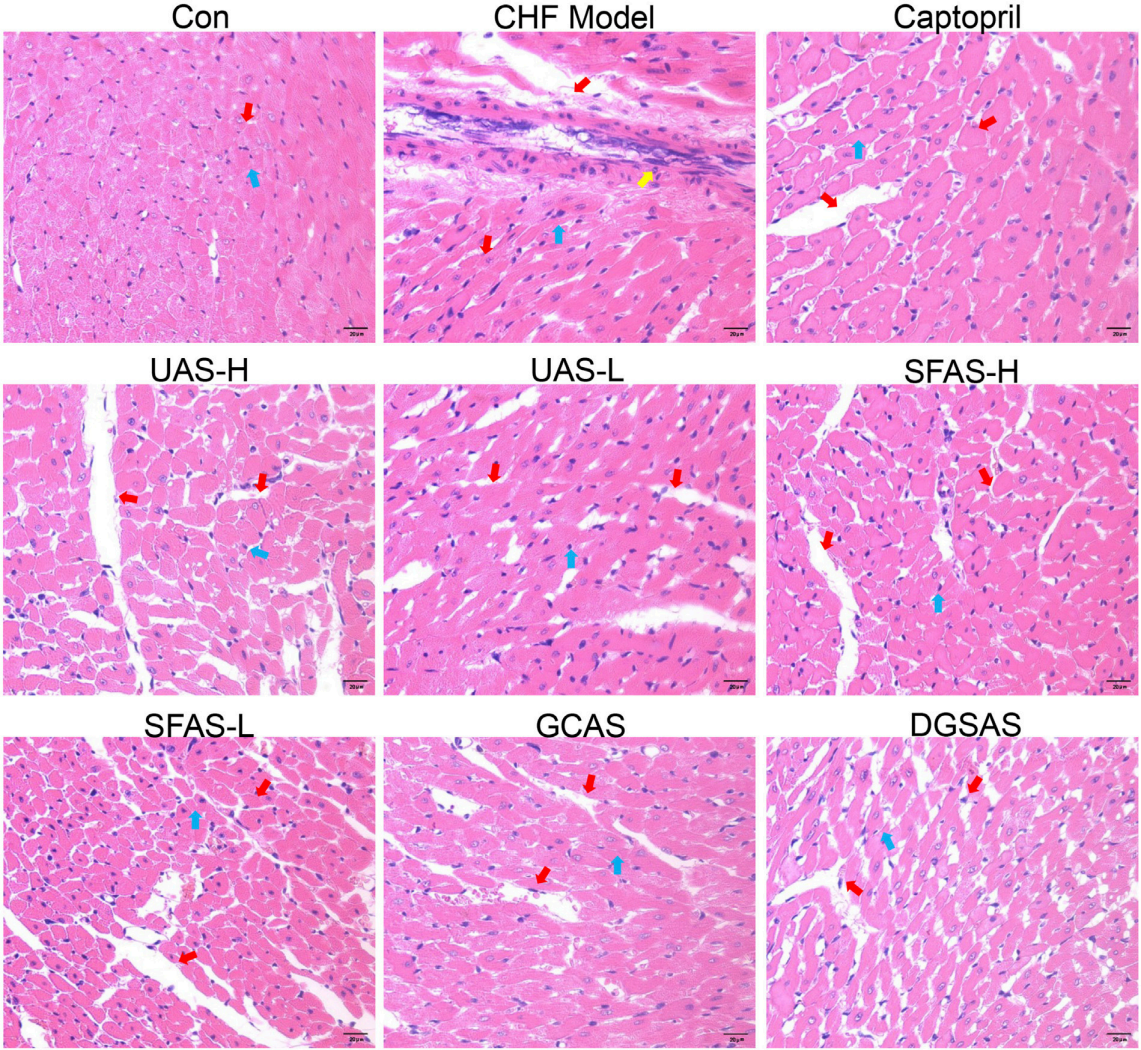
Representative HE staining images of heart tissues in each group (magnification ×400). The red arrows indicate intercellular space. The yellow arrows indicate inflammatory infiltration, and the blue arrows indicate cell and nuclear morphology.

The results showed that the heart tissue of rats in the control group was normal, the myocardial cells were neatly arranged, the structure was clear and compact, and the nuclei were clearly visible. In the CHF model group, the cell arrangement was disordered, the space between the cells increased, cell hypertrophy was distorted, the structure was blurred, the nuclear morphology was abnormal, and there was significant inflammatory cell infiltration. Compared with the CHF model group, cardiomyocytes in the captopril group, UAS-H, SFAS-H, SFAS-L, GCAS, and DGSAS groups were arranged in a more orderly manner, the spacing between cells was reduced, the cell damage was improved, inflammatory cell infiltration was reduced, and cardiomyocyte damage in the captopril group and FAP-H group was significantly reduced (Figure 6).

#### 4.3.2 Masson’s trichrome staining

**FIGURE 7:**
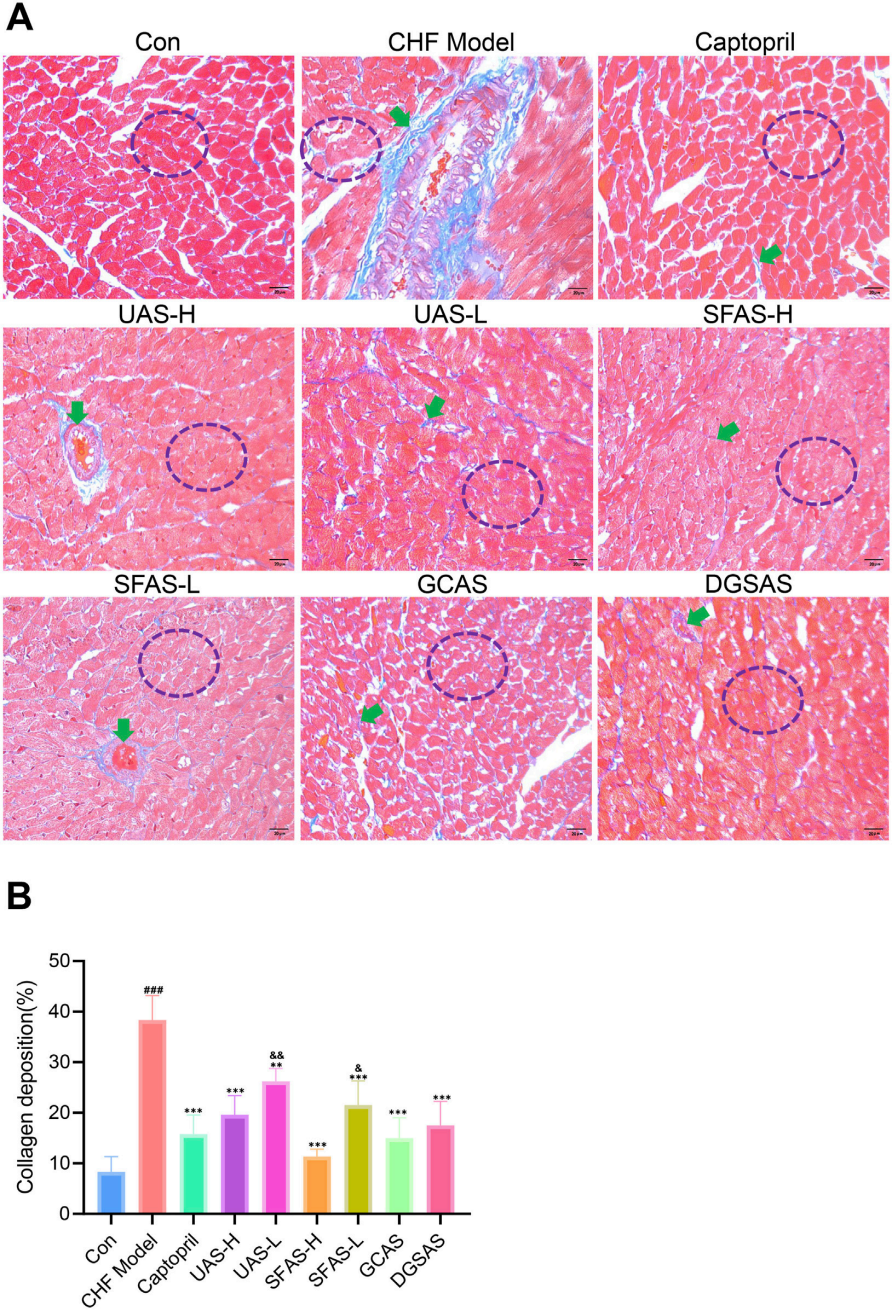
Pathological sections of the hearts of rats in each group. **(A)** Representative Masson staining images of heart tissues in each group (magnification ×400). **(B)** Collagen deposition (*n* = 3). ^###^ *P* < 0.001 in comparisons with the control group; ^**^ *P* < 0.01 and ^***^ *P* < 0.001 in comparisons with the CHF model group; ^&^ *P* < 0.05 and ^&&^ *P* < 0.01 in comparisons with the SFAS-H group. Green arrows indicate collagen deposition. Purple circles indicate cardiac muscle fiber arrangement.

The results showed that the myocardial fibers in the control group were neatly arranged, the transverse lines were clear, and there was no obvious blue collagen fiber deposition. In the CHF model group, the myocardial fibers were disorganized and loose, and interstitial collagen fibers obviously increased, and many blue collagen fibers were deposited (Figure 7A). Compared with the control group, collagen deposition was significantly increased (*P* < 0.001) in the CHF model group (Figure 7B). Compared with the CHF model group, the pathological characteristics of myocardial fibers in each drug administration group were significantly improved, and collagen deposition was significantly reduced (*P* < 0.01 or *P* < 0.001; Figure 7B). Additionally, the arrangement of muscle fibers in the captopril and SFAS-H groups was more regular, with occasional blue collagen fiber deposition, and myocardial fibrosis was significantly improved (Figure 7A).

### 4.4 SFAS alleviated myocardial tissue injury in CHF rats

**FIGURE 8:**
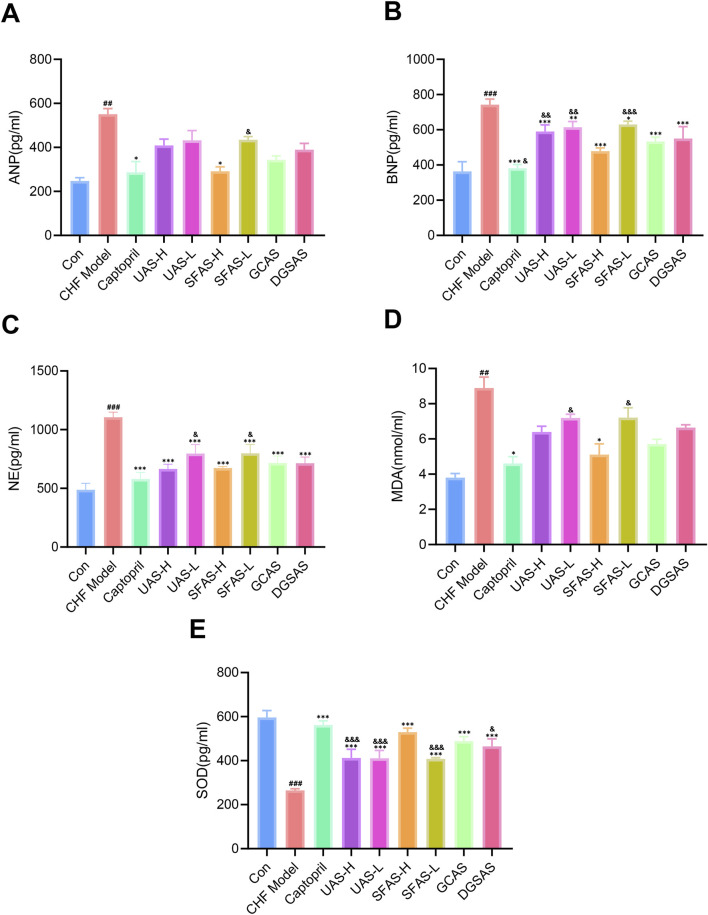
Effects of captopril and different processed aconite products on myocardial injury and oxidative stress in rats with CHF. **(A)** ANP levels in myocardial tissue of rats in each group (*n* = 3). **(B)** BNP levels in myocardial tissue of rats in each group (*n* = 3). **(C)** NE levels in the serum of rats in each group (*n* = 4). **(D)** MDA levels in myocardial tissue of rats in each group (n = 3). **(E)** SOD activity in myocardial tissue of rats in each group (*n* = 3). ^##^ *P* < 0.01 and ^###^ *P* < 0.001 in comparisons with the control group; ^*^ *P* < 0.05, ^**^ *P* < 0.01, and ^***^ *P* < 0.001 in comparisons with the CHF model group; ^&^ *P* < 0.05, ^&&^ *P* < 0.01, and ^&&&^ *P* < 0.001 in comparisons with the SFAS-H group.

Compared with the control group, ANP, BNP, and NE levels in the CHF model group were significantly higher (*P* < 0.01 or *P* < 0.001; Figures 8A–C). Compared with the CHF model group, the ANP level in the captopril and SFAS-H groups was significantly decreased (*P* < 0.05; Figure 8A), and BNP and NE levels in all treatment groups significantly decreased (*P* < 0.05, *P* < 0.01, or *P* < 0.001; Figures 8B,C). Additionally, the ANP levels in the UAS-L group, BNP levels in the UAS-H, UAS-L, and SFAS-L groups, and NE levels in the UAS-L and SFAS-L groups were significantly higher than those in the SFAS-H group, and the BNP levels in the captopril group were significantly lower than those in the SFAS-H group (*P* < 0.05, *P* < 0.01, or *P* < 0.001; Figures 8A–C). These results indicate that while the efficacy of SFAS-H was slightly lower than that of captopril in some respects, it could also effectively reduce the pressure load on the myocardium of male CHF rats and delay ventricular remodeling.

### 4.5 SFAS reduced oxidative stress in the myocardium of CHF rats

Compared with the control group, the MDA level in the myocardial tissue of rats in the CHF model group was significantly higher (*P* < 0.01; Figure 8D), and the SOD level in the myocardial tissue of rats in the CHF model group was significantly lower (*P* < 0.001; Figure 8E). These indicate that the level of oxidative stress in the myocardium was significantly increased. Compared with the CHF model group, the MDA level in the myocardial tissue of rats in the captopril and SFAS-H groups was significantly decreased (*P* < 0.05; Figure 8D), and the SOD level in all administration groups was significantly increased (*P* < 0.001; Figure 8E). Among the groups, the MDA levels in the UAS-L and SFAS-L groups were significantly higher than those of the SFAS-H group, and the SOD levels of the UAS-H, UAS-L, SFAS-L, and DGSAS groups were significantly lower than those of the SFAS-H group (*P* < 0.05 or *P* < 0.001; Figures 8D,E). These results indicate that SFAS-H can improve oxidative stress in CHF rat myocardial tissue. Additionally, its effect is similar to that of captopril, and it has certain advantages over other products.

### 4.6 SFAS increased the activity of respiratory chain enzymes in the myocardium of CHF rats

**FIGURE 9:**
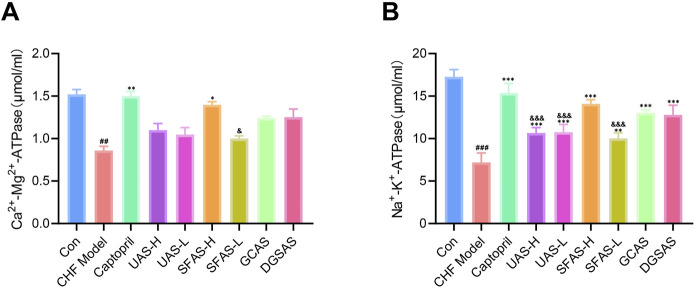
Effects of captopril and different processed aconite products on the activity of respiratory chain enzymes in the myocardial tissue of CHF rats. **(A)** Na^+^-K^+^-ATPase activity in the myocardial tissue of rats in each group (*n* = 3). **(B)** Ca^2+^-Mg^2+^-ATPase activity in the myocardial tissue of rats in each group (*n* = 3). ^##^ *P* < 0.01 and ^###^ *P* < 0.001 in comparisons with the control group; ^*^ *P* < 0.05, ^**^ *P* < 0.01, and ^***^ *P* < 0.001 in comparisons with the CHF model group; ^&^ *P* < 0.05 and ^&&&^ *P* < 0.001 in comparisons with the SFAS-H group.

Compared with the control group, the activities of Na^+^-K^+^-ATPase and Ca^2+^-Mg^2+^-ATPase in the myocardial tissue of rats in the CHF model group were significantly decreased (*P* < 0.01 or *P* < 0.001; Figures 9A,B), indicating that respiratory chain enzymes in the myocardium of CHF rats were significantly decreased and the function of myocardial cells was disrupted. Compared with the model group, Na^+^-K^+^-ATPase activity in rats in each dosing group was significantly increased (*P* < 0.01 or *P* < 0.00; Figure 9A), and Ca^2+^-Mg^2+^-ATPase activities in rats in the captopril and the SFAS-H groups were significantly increased (*P* < 0.05 or *P* < 0.01; Figure 9B). Among the groups, Na^+^-K^+^-ATPase activities in the UAS-H, UAS-L, and SFAS-L groups were significantly lower than those in the SFAS-H group (*P* < 0.001; Figure 9A), and the Ca^2+^-Mg^2+^-ATPase activity in the SFAS-L group was significantly lower than that in the SFAS-H group (*P* < 0.05; Figure 9B).

### 4.7 SFAS improves mitochondrial pathological state in CHF rats

**FIGURE 10:**
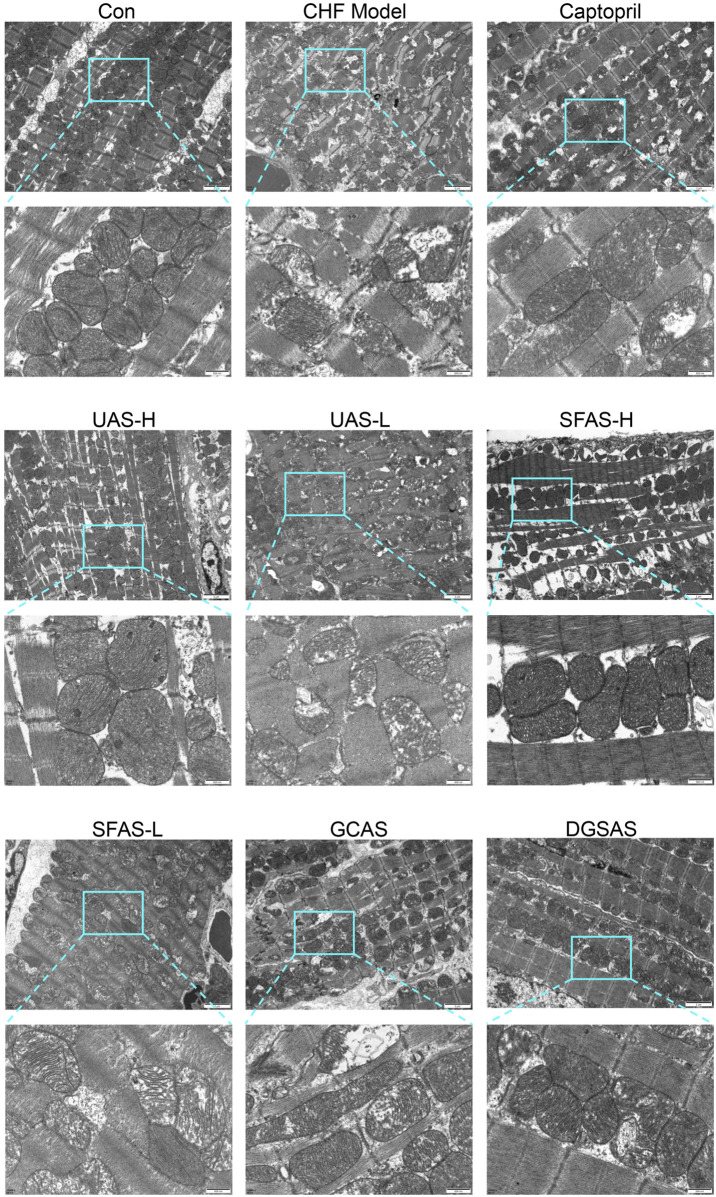
The ultrastructure of myocardial mitochondria in each group of rats was observed by transmission electron microscopy.

Electron microscopic examination of the heart showed that the mitochondria of rats in the control group were neatly arranged in the myocardial tissue, with regular shape, dense and parallel arrangement of cristae structure, and a uniform matrix. Compared with the control group, the arrangement of myocardial mitochondria in CHF model rats was disordered, the number was slightly reduced, the volume of mitochondria was increased, the structure was damaged, the matrix cavity was expanded, the distance between the inner and outer membranes was widened, mitochondrial cristae were fragmented or absent, and internal vacuolization was evident. Compared with the CHF model group, the pathological state of myocardial mitochondria in rats of the captopril, UAS-H, SFAS-H, GCAS, and DGSAS groups improved, the mitochondrial arrangement was relatively orderly, the mitochondrial structure was relatively intact, the damage to cristae was reduced, and the swelling or vacuolation was improved. Furthermore, the number of mitochondria was further reduced in the UAS-L and SFAS-L groups, and the pathological state persisted (Figure 10).

### 4.8 SFAS affects FFA transport and energy production in the myocardium of CHF rats

**FIGURE 11:**
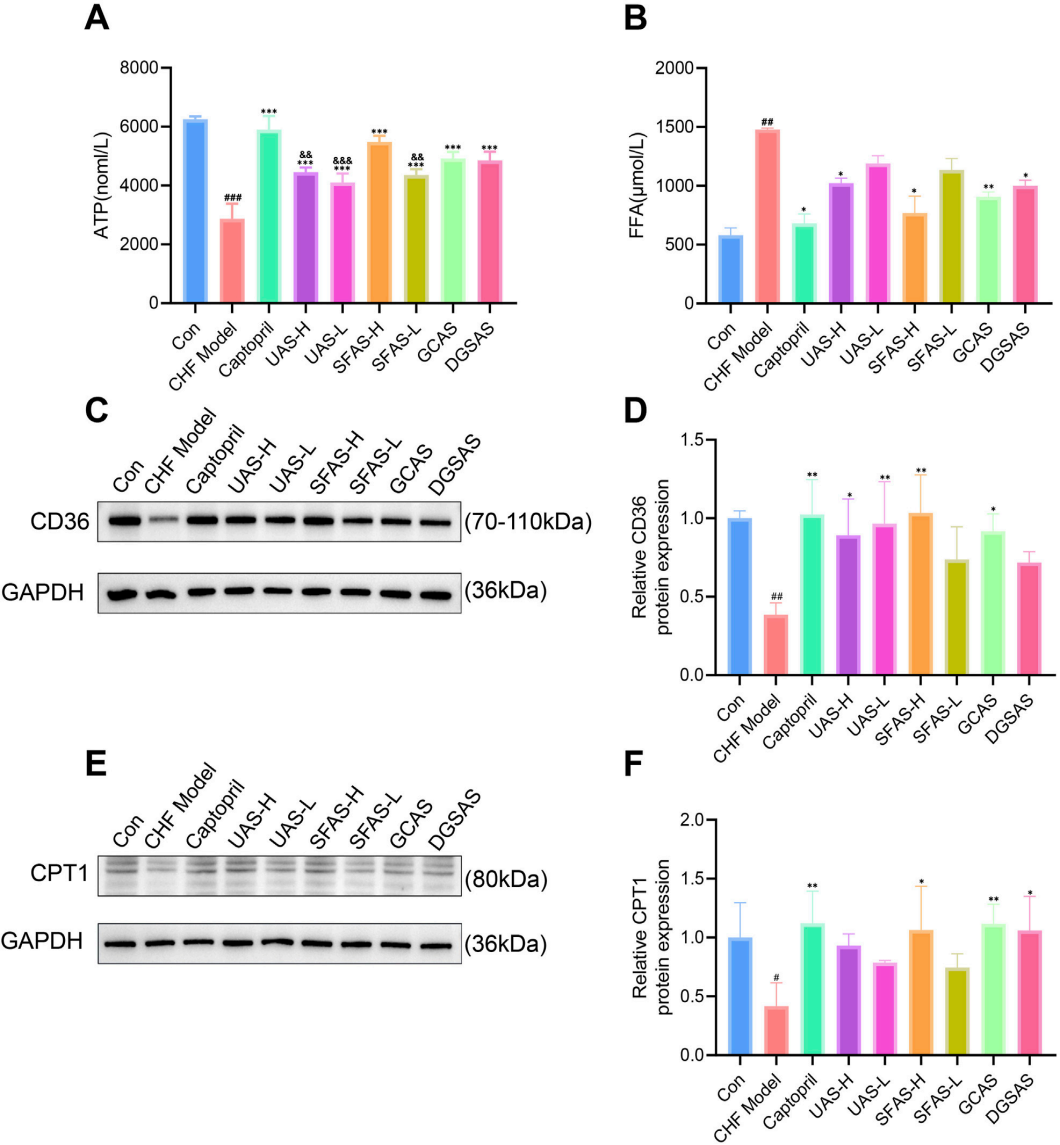
Effects of captopril and different processed aconite products on FFA transport and energy metabolism in the myocardial tissue of CHF rats. **(A)** ATP concentration in myocardial tissue of rats in each group (*n* = 3). **(B)** FFA levels in myocardial tissue of rats in each group (*n* = 3). **(C)** CD36 Western blot images. **(D)** Relative CD36 protein expression in myocardial tissue of rats in each group (*n* = 3). **(E)** CPT1Western blot images. **(F)** Relative CPT1 protein expression in myocardial tissue of rats in each group (*n* = 3). ^#^ *P* < 0.05, ^##^ *P* < 0.01, and ^###^ *P* < 0.001 in comparisons with the control group; ^*^ *P* < 0.05, ^**^ *P* < 0.01, and ^***^ *P* < 0.001 in comparisons with the CHF model group; ^&&^ *P* < 0.01 and ^&&&^ *P* < 0.001 in comparisons with the SFAS-H group.

Compared with the control group, the ATP concentration in the myocardial tissue of rats in the CHF model group was significantly decreased (*P* < 0.001), FFA levels were significantly increased (*P* < 0.01; Figures 11A,B), and CD36 and CPT1 protein expressions were significantly decreased (*P* < 0.05 or *P* < 0.01; Figures 11C–F), indicating impaired FFA transport and dysregulated energy metabolism in the myocardium of CHF rats. Compared with the CHF model group, the ATP content in all drug administration groups was significantly increased (*P* < 0.001; Figure 11A); FFA levels in the captopril, UAS-H, SFAS-H, GCAS, and DGSAS groups were significantly decreased (*P* < 0.05 or *P* < 0.01; Figure 11B); CD36 expression levels were significantly increased in the captopril, UAS-H, UAS-L, SFAS-H, and GCAS groups (*P* < 0.05 or *P* < 0.01; Figures 11C,D); and CPT1 expression levels in the captopril, SFAS-H, GCAS, and DGSAS groups were significantly increased (*P* < 0.05 or *P* < 0.01; Figures 11E,F). Among the groups, ATP generation in the UAS-L, UAS-H, and SFAS-L groups was significantly lower than that in the SFAS-H group (*P* < 0.01 or *P* < 0.001; Figure 11A). These results indicate that SFAS-H enhanced FFA transport and ATP production, similar to captopril.

### 4.9 SFAS affects AMPK-PGC-1α-SIRT3 pathway activity in the myocardium of CHF rats

**FIGURE 12:**
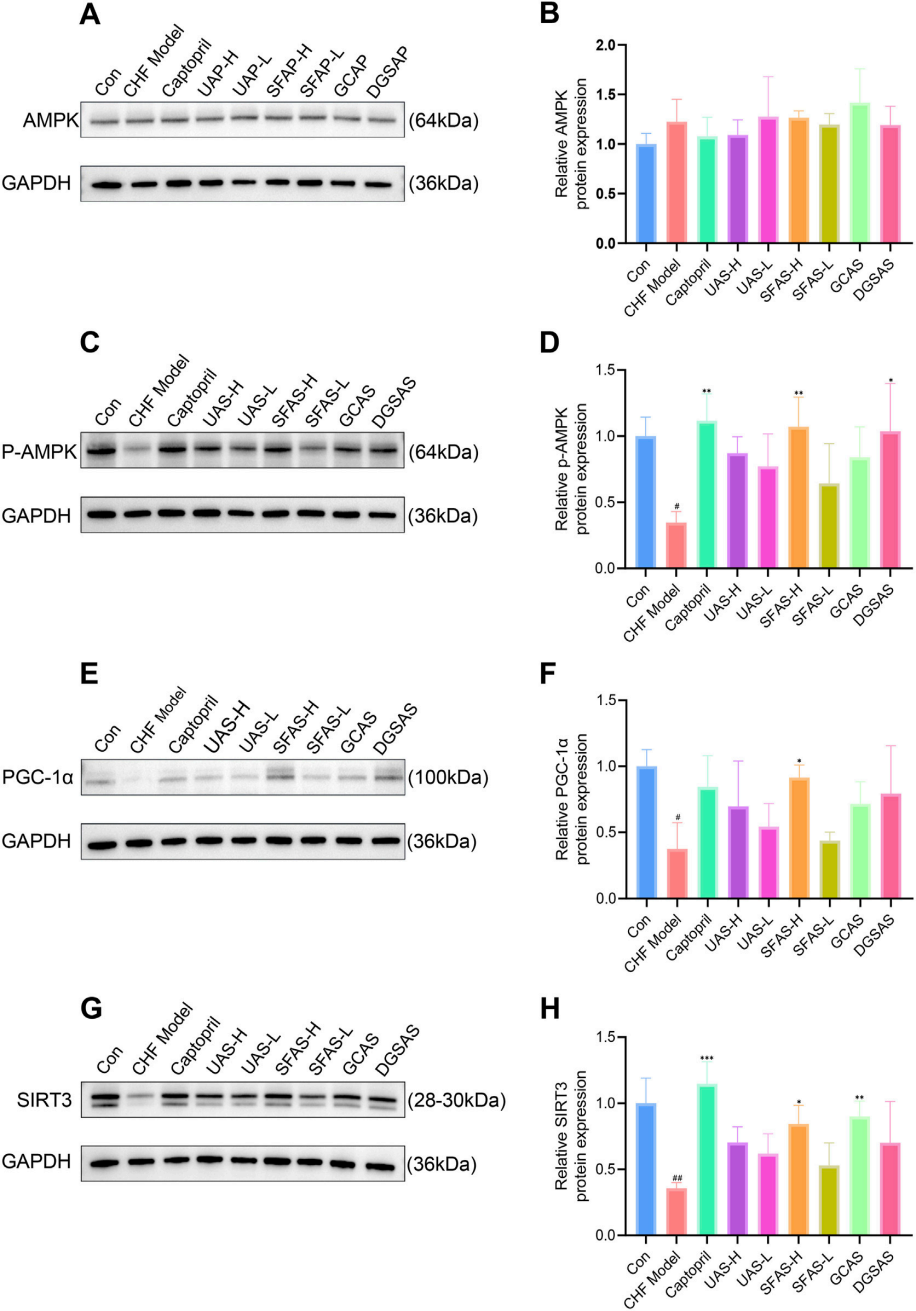
Effects of captopril and different processed aconite products on the AMPK, PGC-1α, and SIRT3 pathway in the myocardial tissue of CHF rats. **(A)** AMPK Western blot images. **(B)** Relative AMPK protein expression in the myocardial tissue of rats in each group (*n* = 3). **(C)** p-AMPK Western blot images. **(D)** Relative p-AMPK protein expression in the myocardial tissue of rats in each group (*n* = 3). **(E)** PGC-1α Western blot images. **(F)** Relative PGC-1α protein expression in the myocardial tissue of rats in each group (*n* = 3). **(G)** SIRT3 Western blot images. **(H)** Relative SIRT3 protein expression in the myocardial tissue of rats in each group (*n* = 3). ^#^ *P* < 0.05 and ^##^ *P* < 0.01 in comparisons with the control group; ^*^ *P* < 0.05, ^**^ *P* < 0.01, and ^***^ *P* < 0.001 in comparisons with the CHF model group.

No significant differences in total AMPK protein expression were observed among groups. However, when compared with the control group, p-AMPK, PGC-1α, and SIRT3 expression levels were significantly decreased in the CHF model group (*P* < 0.05 or *P* < 0.01; Figures 12A–H). Compared with the CHF model group, p-AMPK, PGC-1α, and SIRT3 expression in all groups increased to different degrees. Specifically, p-AMPK expression was significantly increased in the captopril, SFAS-H, and DGSAS groups (*P* < 0.05 or *P* < 0.01; Figures 12C,D), PGC-1α expression significantly increased in the SFAS-H group (*P* < 0.05; Figures 12E,F), and SIRT3 expression significantly increased in the captopril, SFAS-H, and GCAS groups (*P* < 0.05, *P* < 0.01, or *P* < 0.001; Figures 12G,H). These results indicate that SFAS-H has a strong regulatory effect on the AMPK/PGC-1α/SIRT3 pathway, which controls cardiac energy metabolism in CHF rats.

## 5 Discussion

CHF is a complex clinical syndrome of multifactorial etiology. Previous studies have shown that its pathogenesis mainly includes ventricular remodeling, overactivation of the nervous and humoral systems, and impaired energy metabolism in cardiomyocytes. Among these factors, impaired energy metabolism in cardiomyocytes is a key factor in the occurrence and progression of heart failure. DOX, an anthracycline antibiotic widely used to treat various cancers (e.g., breast and bladder cancers), is associated with dose-limiting cardiotoxicity that can culminate in CHF. Due to its strong cardiotoxicity, large doses of DOX can cause CHF. Existing studies have shown that cardiotoxicity is closely related to mitochondrial damage (Catanzaro et al., 2019; Timm and Tyler, 2020). Based on this, we used DOX to establish the rat CHF model in this study.

In this study, the rat CHF model was established by intraperitoneal injection of DOX. When the cumulative dose of DOX reached 18 mg/kg, the rats in the CHF model group showed obvious heart failure manifestations such as weight loss, significant decrease in activity level, and ascites. Ultrasonography revealed that the LVEF and LVFS were significantly lower than those in the control group. The results of the cardiac pathological section showed that the space between myocardial cells in the CHF model rats was increased, the myocardial structure was distorted, the nuclear morphology was abnormal, inflammatory cell infiltration was obvious, myocardial fibers were disorganized and loose, interstitial collagen fibers were increased, and a large amount of blue collagen fibers could be seen. Additionally, ANP, BNP, MDA, and NE levels in the CHF model group significantly increased, while SOD expression was significantly decreased. The above results suggest that oxidative stress significantly increased in the cardiac cells of rats after the cumulative dose of DOX administered by intraperitoneal injection reached 18 mg/kg. Furthermore, the myocardial mitochondria were damaged and mitochondrial dysfunction occurred, leading to myocardial cell injury, ventricular remodeling, cardiac function decline, and ultimately CHF.

A previous work by our group demonstrated that SFAS’s anti-CHF effects involve mitigating inflammation, suppressing renin-angiotensin-aldosterone system (RAAS) overactivity, modulating adrenergic receptor expression, and improving myocardial energy metabolism (Yu, 2020). Therefore, this study used myocardial energy metabolism as the starting point to investigate the mechanism underlying the effectiveness of SFAS in treating CHF. The results showed that SFAS-H could increase LVEF and LVFS, improve hypertrophy and necrosis of cardiomyocytes and myocardial fibrosis, and reduce cardiac ANP, BNP, NE, and MDA levels in CHF rats. Furthermore, it controls oxidative stress in cardiomyocytes and improves cardiac function by increasing SOD levels.

Na^+^-K^+^-ATPase and Ca^2+^-Mg^2+^-ATPase are transmembrane ion pumps that are critical for maintaining cellular ionic homeostasis and mitochondrial membrane potential. They play important roles in maintaining cell function and mitochondrial membrane stability and are indispensable in cardiac energy metabolism. When the oxidative stress increases in myocardial cells, Na^+^-K^+^-ATPase and Ca^2+^-Mg^2+^-ATPase activities decrease, cell function decreases, mitochondrial inner membrane permeability is destroyed, the ion concentration gradient inside and outside the mitochondrial membrane changes, and the energy metabolism process is unbalanced (Abdulkareem et al., 2019; Dhalla et al., 2024). Therefore, to explore the effect of SFAS on the activity of cardiac respiratory streptokinase, we measured the concentration of Na^+^-K^+^-ATPase and Ca^2+^-Mg^2+^-ATPase. The results showed that SFAS-H could significantly increase Na^+^-K^+^-ATPase and Ca^2+^-Mg^2+^-ATPase concentration in the myocardial tissue of CHF rats, improve cell function, enhance mitochondrial membrane stability, and alleviate myocardial mitochondrial damage.

Mitochondria are the primary sites of cellular energy metabolism, with the cristae harboring the protein complexes essential for oxidative phosphorylation. The mitochondrial cristae matrix contains ATP synthase, which uses the energy generated by the respiratory chain to synthesize ATP (Cogliati et al., 2016). Therefore, during energy metabolism in the myocardium, the number of mitochondria, the density of mitochondrial cristae, and the integrity of mitochondrial structure are closely related to the amount of ATP produced. FFA are the main substrates for normal heart energy metabolism. FFAs are transported into cardiomyocytes through CD36, activated as fatty acyl-Coenzyme A in cytosols, and then catalyzed by CPT1 and other transport proteins, they are transported into mitochondria for β oxidation, completing the tricarboxylic acid cycle (Azevedo et al., 2013; Bairwa et al., 2016). In advanced heart failure, the cardiac energy metabolism process is transformed (Stanley et al., 2005). On the one hand, the number of myocardial mitochondria decreases, and the density of cristae in surviving mitochondria decreases under oxidative stress, reducing oxidative phosphorylation and the ATP production capacity. On the other hand, the expression of the intracellular transport proteins CD36 and CPT1 decreases, resulting in the failure of FFA transport into the mitochondrial matrix, accumulation of FFAs outside the mitochondria, and insufficient concentration of substrates inside the mitochondria to supply its energy production process, resulting in reduced energy production. After administration of SFAS-H to CHF rats, the number of mitochondria and the cristae density in rats partially recovered, CD36 and CPT1 activities significantly increased, FFA accumulation was reversed, ATP production increased, and fatty acid oxidation significantly improved.

The AMPK/PGC-1α/SIRT3 axis constitutes a key energy-sensing and regulatory pathway, and it plays an important role in mitochondrial energy metabolism and other processes. AMPK is a key molecule in the regulation of biological energy metabolism and is considered a “metabolic switch,” playing a pivotal role in regulating cardiac energy metabolism. When cardiac stress responses occur, the intracellular AMP/ATP ratio increases, and AMPK is activated and phosphorylated, producing p-AMPK. p-AMPK acts downstream. At this time, the ATP pathway is activated to inhibit ATP consumption in tissues, promote ATP production, and maintain cardiac energy metabolism balance (Bairwa et al., 2016; Yu et al., 2023). PGC-1α is a regulator of mitochondrial biosynthesis, and it is directly regulated by AMPK. It can maintain and repair cell structure and function through the regulation of mitochondrial production, and is an essential molecule for mitochondrial fatty acid metabolism (Xu et al., 2024; You et al., 2024). The downstream signal of PGC-1α, SIRT3, is an NAD + -dependent deacetylase. It is mainly expressed in mitochondria and is involved in regulating the mitochondrial energy metabolism in cardiomyocytes. It can mediate the myocardial protective effect of PGC-1α and can also work with AMPK to maintain energy homeostasis, protect cardiomyocytes from oxidative damage and aging, and inhibit cardiac hypertrophy (Afzaal et al., 2022; Silaghi et al., 2021; Xin and Lu, 2020). A large body of research indicates that the AMPK-PGC-1α-SIRT3 pathway is crucial for the prevention and treatment of heart disease. For example, a study has pointed out that luteolin can prevent DOX-induced myocardial cell damage through the mediation of the AMPK/SIRT3/Nrf2 pathway (Zou et al., 2024). Another study has shown that naringin can promote mitochondrial generation and relieve mitochondrial oxidative stress by activating AMPK/PGC-1α, thus having an anti-myocardial ischemia-reperfusion injury effect (Yu et al., 2019). Moreover, a study demonstrated that melatonin can reduce mitochondrial oxidative stress while enhancing mitochondrial biogenesis to protect mitochondrial function, ultimately alleviating MI/R injury in type 1 diabetes via the AMPK-PGC1α-SIRT3 axis (Yu et al., 2017).

To date, no studies have specifically investigated the involvement of the AMPK/PGC-1α/SIRT3 pathway in the anti-CHF mechanisms of aconitum and its processed products. Based on this, this study evaluated changes in AMPK/PGC-1α/SIRT3 pathway activities, changes in myocardial energy metabolism, and the relevant mechanisms in CHF rats treated with SFAS. The expression levels of related protein molecules were determined by Western blot, and the results showed that p-AMPK, SIRT3, and PGC-1α protein levels significantly increased after SFAS-H intervention in CHF rats, suggesting that SFAS can protect myocardial mitochondria by activating the AMPK/PGC-1α/SIRT3 pathway, thereby improving heart failure.

Our findings demonstrate that SFAS intervention ameliorates CHF symptoms and improves cardiac function by regulating the AMPK-PGC-1α-SIRT3 energy metabolism pathway. Specifically, SFAS phosphorylates myocardial AMPK into p-AMPK through the activation of myocardial AMPK, restoring p-AMPK levels and consequently restoring the normal energy metabolism environment in myocardial disorders. Concurrently, SFAS enhances PGC-1α and SIRT3 expression in the myocardium, thereby restoring mitochondrial synthesis to normal levels. This supplements the number of mitochondria in the myocardium, making the energy supply of the myocardium sufficient to support the maintenance and repair of the structure and function of damaged myocardial cells, reducing oxidative stress in myocardium and mitochondria, improving the level of mitochondrial biogenesis, and rebuilding the steady state of myocardial energy metabolism while preventing further oxidative damage to myocardial cells and their mitochondria.

**FIGURE 13:**
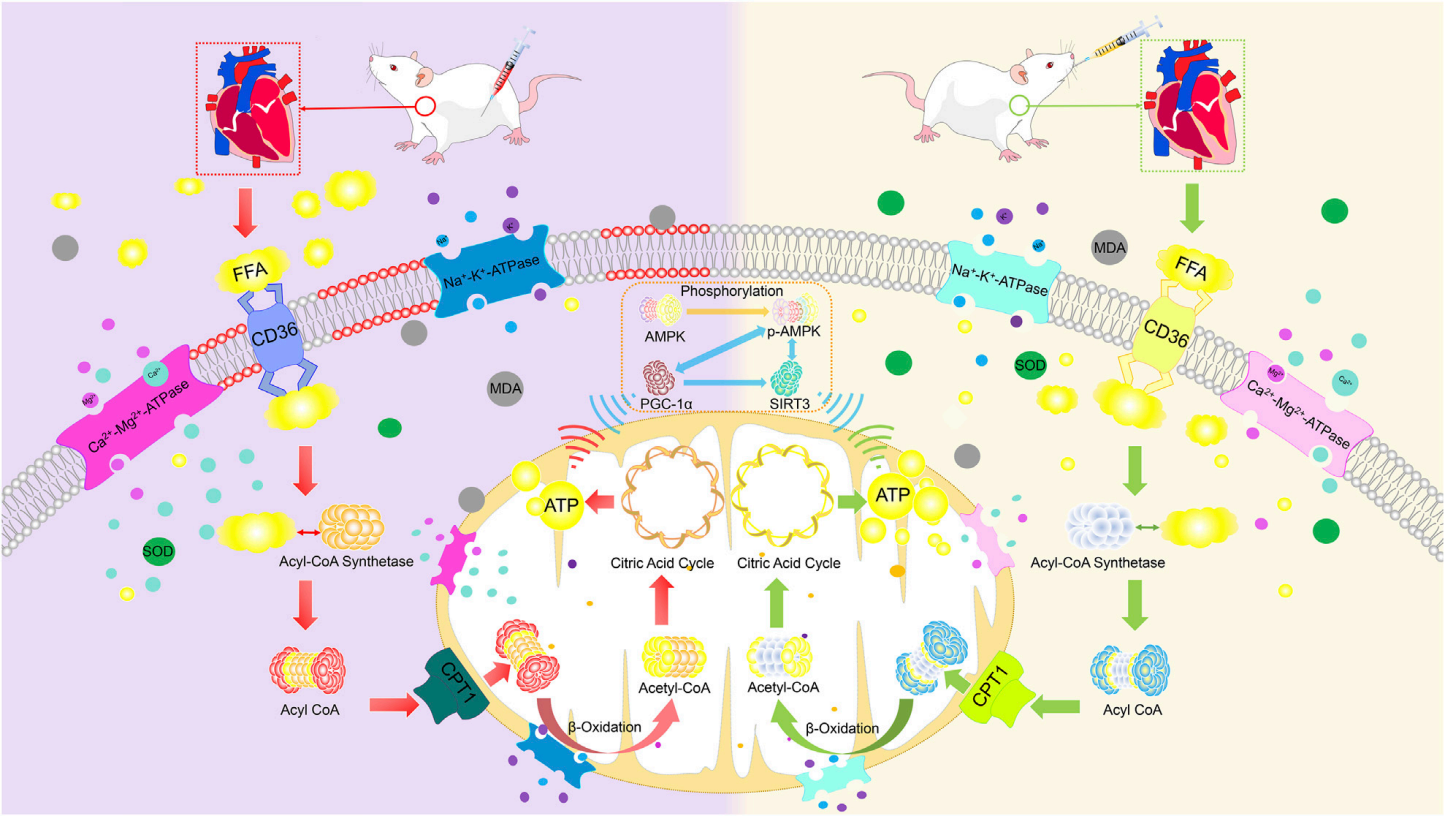
SFAS improves DOX-induced CHF via the AMPK-PGC-1α-SIRT3 pathway.

This study demonstrated that SFAS promotes the expression of PGC-1α and SIRT3 in the heart by activating AMPK phosphorylation, restores the number of mitochondria, achieves preliminary recovery of energy metabolism homeostasis in the heart, and promotes the transport of FFAs into mitochondria by coordinating the activation of CD36, enhancing CPT1 expression, and improving Na^+^-k^+^-ATPase and Ca^2+^-Mg^2+^-ATPase activities to increase mitochondrial ATP production capacity, thereby reducing MDA levels and increasing SOD levels in the heart. This prevents further damage to myocardial cells and mitochondria, thereby reducing ANP, BNP, and NE levels. Essentially, it restores energy metabolism homeostasis in the heart, delays ventricular remodeling, reduces myocardial injury, and restores heart function (Figure 13).

Processing (Paozhi) is a distinctive pharmaceutical technology in TCM. It features a sophisticated system with diverse methods that can alter drug properties, reduce toxicity, and enhance efficacy. Specific processing methods are selected based on the intended therapeutic purpose and target disease.

This study investigated the anti-heart failure mechanism of SFAS and compared its efficacy against that of other processed aconite products. UAS, DGSAS, and GCAS were selected for comparison. UAS is a simple gun product with strong toxicity, and it generally requires further processing; GCAS is a standard gun product according to the Pharmacopoeia of the People’s Republic of China. SFAS and DGSAS originate from Jiangchang gang. Previous studies by our research group have shown that both have good therapeutic effects on CHF, and their therapeutic effects on CHF rats differ based on sex (Yu, 2020).

For dosing, we converted the maximum dosage of aconite (15 g) according to the Pharmacopoeia of the People’s Republic of China to obtain the high dose. By examining high- and low-SFAS and UAS dose groups, we discovered that the high dose had better efficacy, and a comparison of the same doses of different aconite products revealed that SFAS, DGSAS, and GCAS were superior to UAS. This may be because UAS was produced by performing only two processes (bleaching and cutting), and there was no high-temperature processing, such as steaming, boiling, and frying, to convert biester alkaloids to single ester alkaloids. Additionally, this may be caused by the short frying time, demonstrating the influence of complex processing technology on the curative effect of aconite and the convenience of administering the medication. Furthermore, different complex processing methods can give the same medicine different curative effects. Regarding the prepared aconite products in this study, DGSAS involves a long steaming process and GCAS involves a long cooking process, while SFAS uses high-temperature sand frying and a short cooking time, which may lead to different changes in the contents of the various alkaloid components, resulting in different therapeutic effects.

In this study, SFAS-H had particularly potent effects on the AMPK/PGC-1α/SIRT3 pathway, with associated improvements in mitochondrial function and energy metabolism in male CHF rats. Given the documented clinical preferences within the Jianchang gang tradition (SFAS for males and DGSAS for females) and the urgent need for new CHF treatments, these findings provide a molecular basis supporting the exploration of SFAS and potentially other Jianchang gang processed aconites, such as those used in Zhenwu decoction, for CHF management. However, this study has some limitations. First, this study focused exclusively on fatty acid oxidation; glucose metabolism pathways (e.g., PI3K/Akt/GLUT) were not evaluated, potentially limiting the comprehensiveness of the elucidated mechanisms. Second, the comparison of the efficacy of the different products can only explain the therapeutic advantages of SFAS over GCAS, as only male rats were studied. Consequently, potential sex-specific effects or the rationale for the documented clinical sex preferences for SFAS for males versus DGSAS for females could not be elucidated. Therefore, future studies should expand investigations to the PI3K/Akt/GLUT signaling pathway and incorporate female rodent models for a comparative analysis of DGSAS efficacy.

## 6 Conclusion

SFAS activated AMPK and triggered its phosphorylation (p-AMPK), initiating a signaling cascade that specifically upregulated PGC-1α and SIRT3. This axis orchestrated mitochondrial biogenesis and enhanced cardiac antioxidant capacity. Critically, the AMPK-PGC-1α-SIRT3 pathway rectified impaired cardiac fatty acid oxidation by increasing CD36 and CPT1 expression, thereby accelerating FFA consumption and boosting ATP synthesis. Concomitantly, it attenuated oxidative stress and mitochondrial structural damage. In summary, SFAS ameliorates CHF by restoring impaired fatty acid oxidation and replenishing ATP via the activation of the AMPK/PGC-1α/SIRT3 signaling pathway, while concurrently suppressing oxidative stress.

## Data Availability

The original contributions presented in the study are included in the article/Supplementary Material, further inquiries can be directed to the corresponding authors.

## Supplementary material

Supplementary Data Sheet 1 SFAS Component Determination

## Glossary

ACN: Aconine
AMPK: AMPactivated protein kinase
ANP: Atrial natriuretic peptide
ATP: Adenosine triphosphate
BAC: Benzoylaconine
BCA: Bicinchoninic acid
BHC: Benzoylhypaconine
BMA: Benzoylmesaconine
BNP: Brain natriuretic peptide
CHF: Chronic heart failure
DGSAS: Dried ginger steamed aconite slices
DOX: Doxorubicin
ECL: Enhanced chemiluminescence
ELISA: Enzymelinked immunosorbent assays
FFA: Free fatty acid
FU: Fuziline
GCAS: Glycyrrhiza cooked aconite slices
H&E: Hematoxylin and Eosin
HC: Hypaconitine
HCN: Hypaconine
LVEF: Left ventricular ejection fraction
LVFS: Left ventricular shortening fraction
MAN: Mesine
MDA: Malondialdehyde
PBS: Phosphatebuffered saline
RAAS: Reninangiotensinaldosterone system
RIPA: Radioimmunoprecipitation assay
SFAS: Sandfired aconite slices
SOD: Superoxide dismutase
TCM: Traditional Chinese medicine
TEM: Transmission electron microscopy
UAS: Unprocessed aconite slices
XIC: Extract ion chromatography
