# Supplementary material for: Improved myocardial mitochondrial energy metabolism in rats with chronic heart failure by modifying fatty acid oxidation using an extract of sand-fired aconite (Jianchang gang processing)
Source: Front Pharmacol. 2025 Sep 11;16:1600410. doi: 10.3389/fphar.2025.1600410 (PMC12461213; doi:10.3389/fphar.2025.1600410)
Supplement: Supplementary file 1 [file DataSheet1.pdf]

UPLC-MS/MS analysis Reference material

RT 3.05:Aconitine (AC)

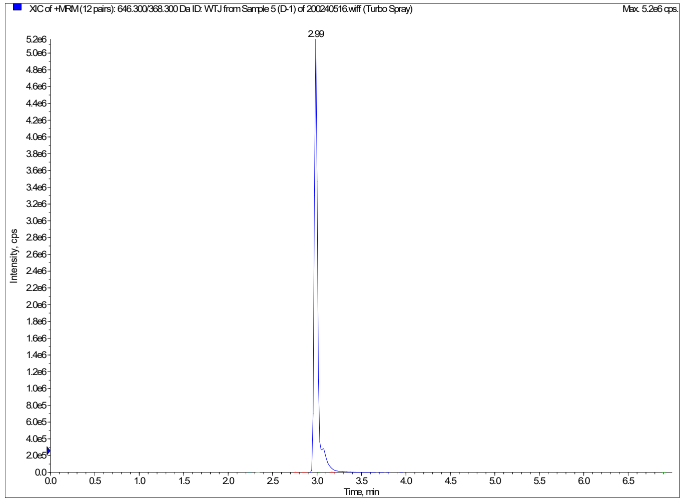

RT 2.89:Mesaconitine (MA)

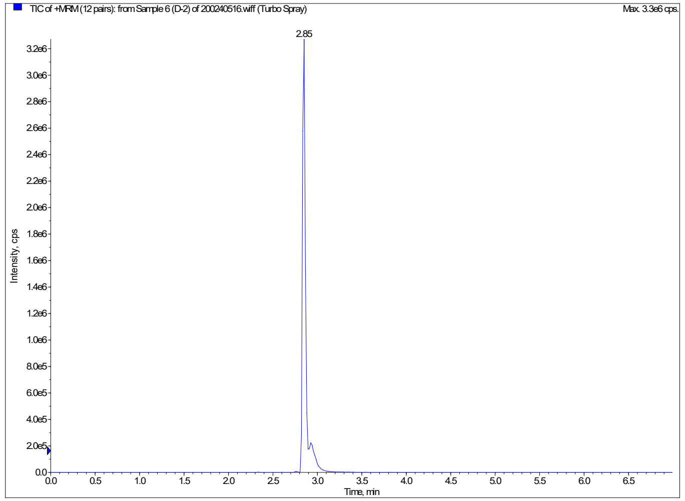

RT 3.08:Hypaconitine (HC)

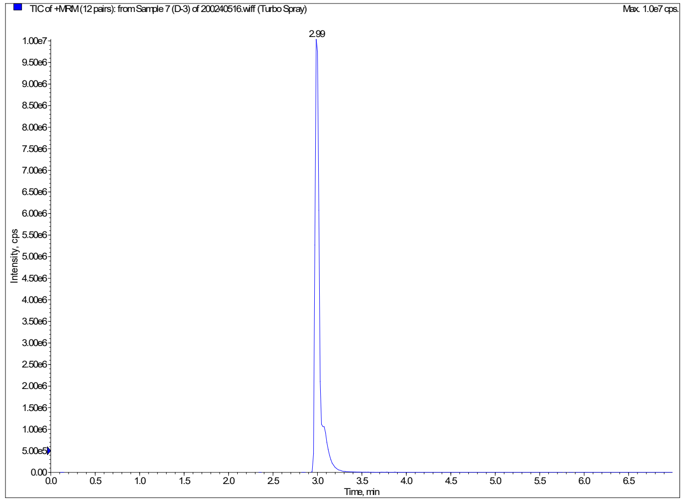

RT 2.68:Benzoylaconine (BAC)

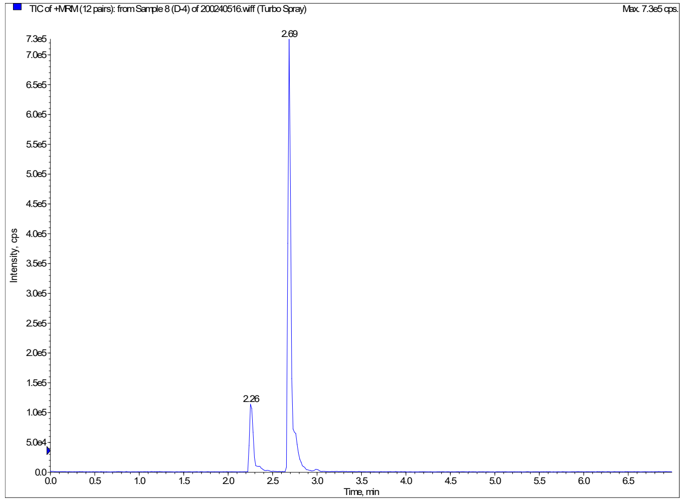

RT 2.58:Benzoylmesaconine (BMA)

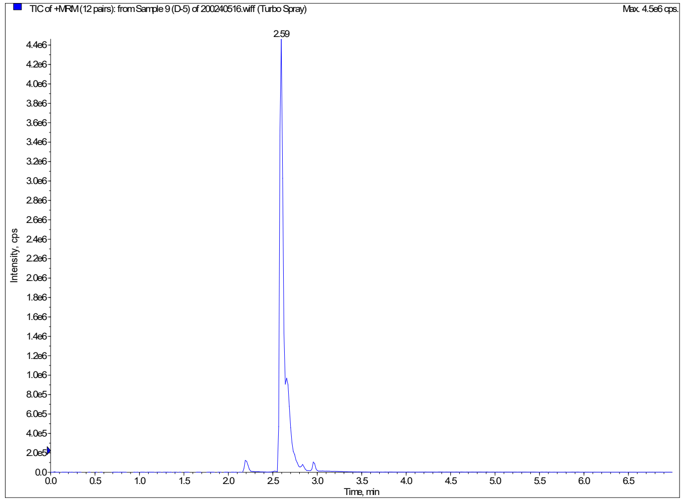

RT 2.73:Benzoylhypaconine (BHC)

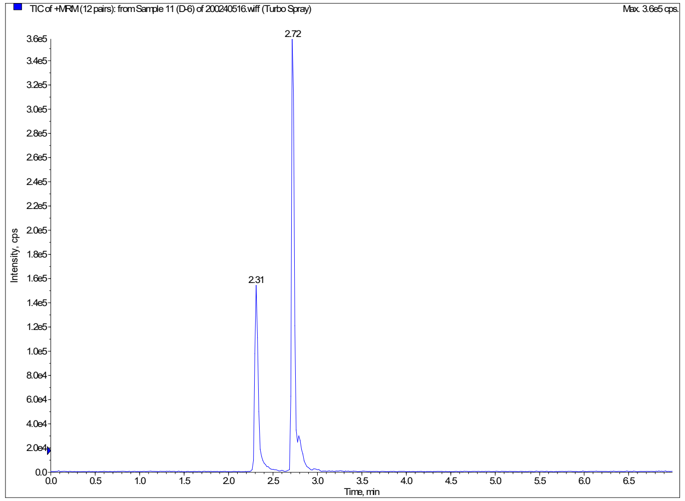

UPLC-MS/MS analysis Reference material

RT 2.18:Aconine (ACN)

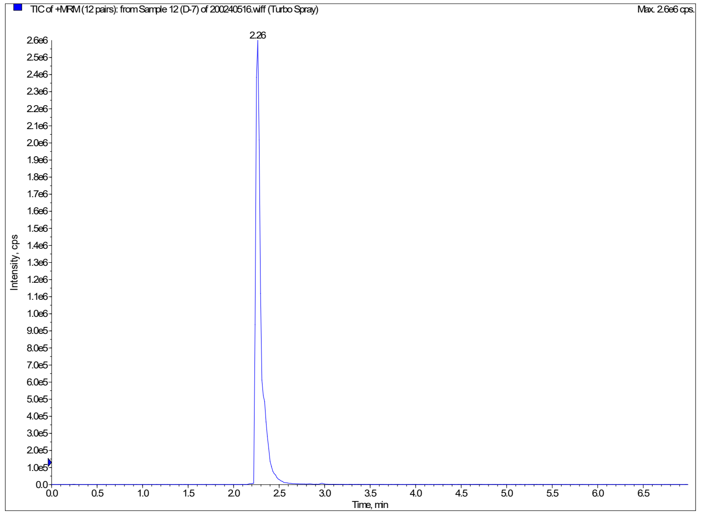

RT 2.13:Mesaconine (MAN)

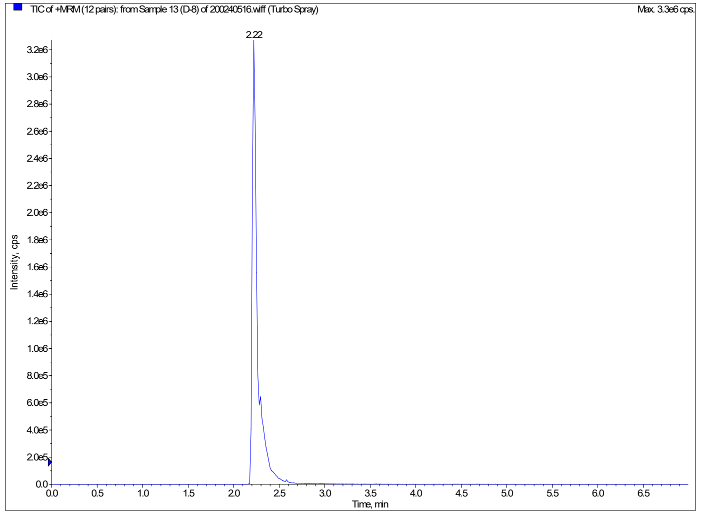

RT 2.22:Hypaconine (HCN)

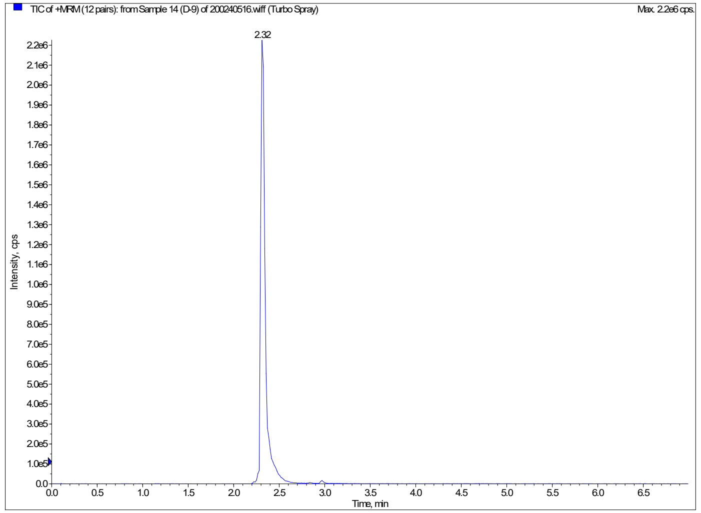

RT 2.23:Fuziline (FU)

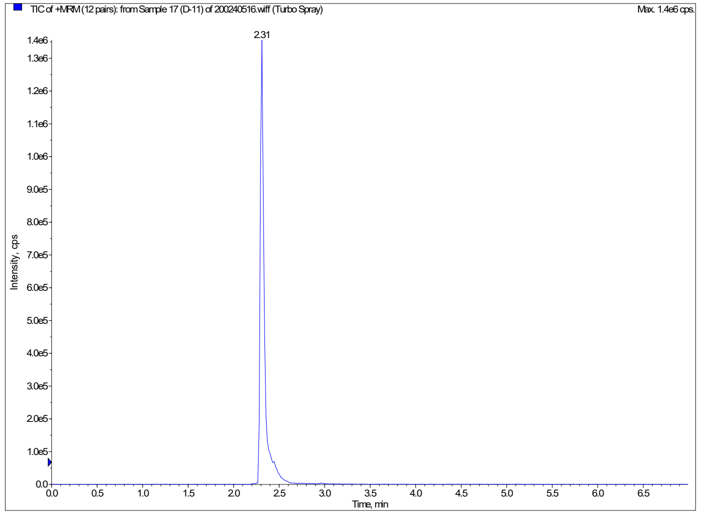

RT 2.17:Higenamine (HI)

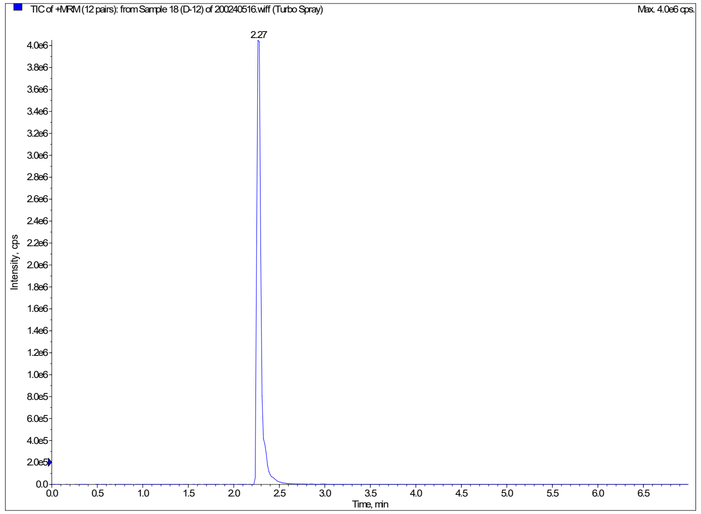

UPLC-MS/MS analysis

SFAP

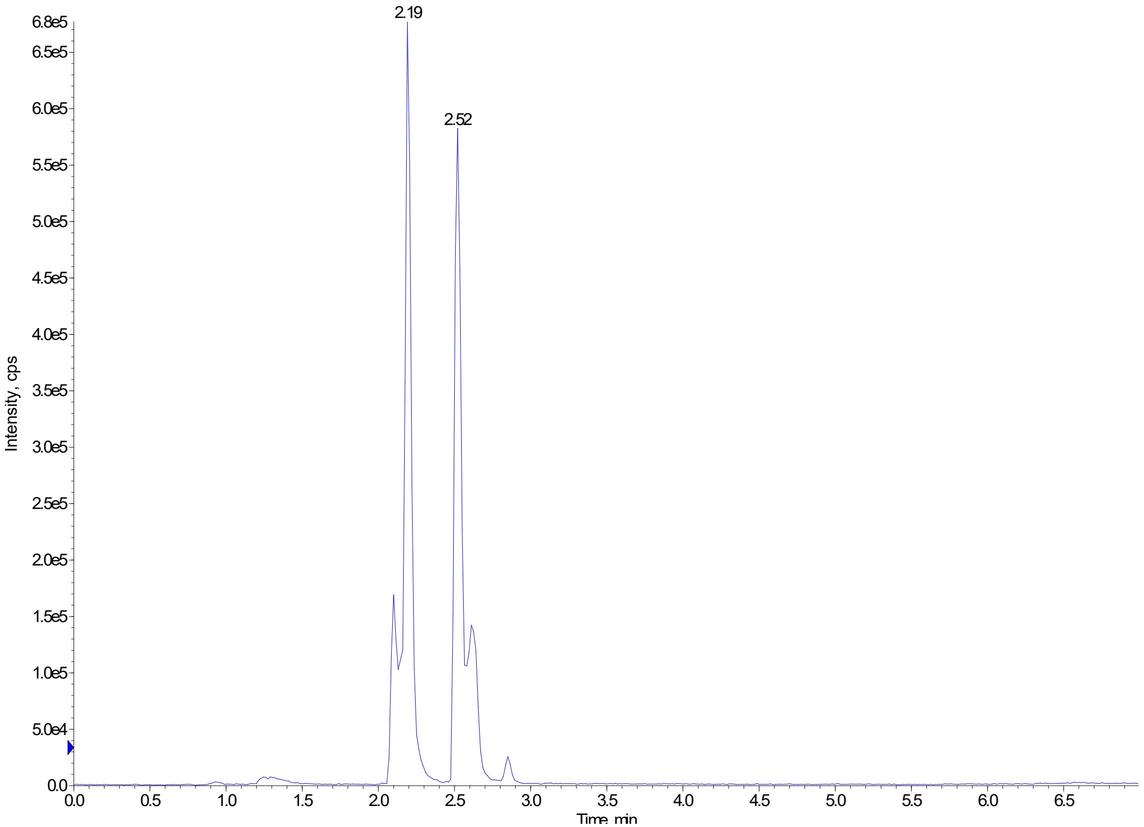

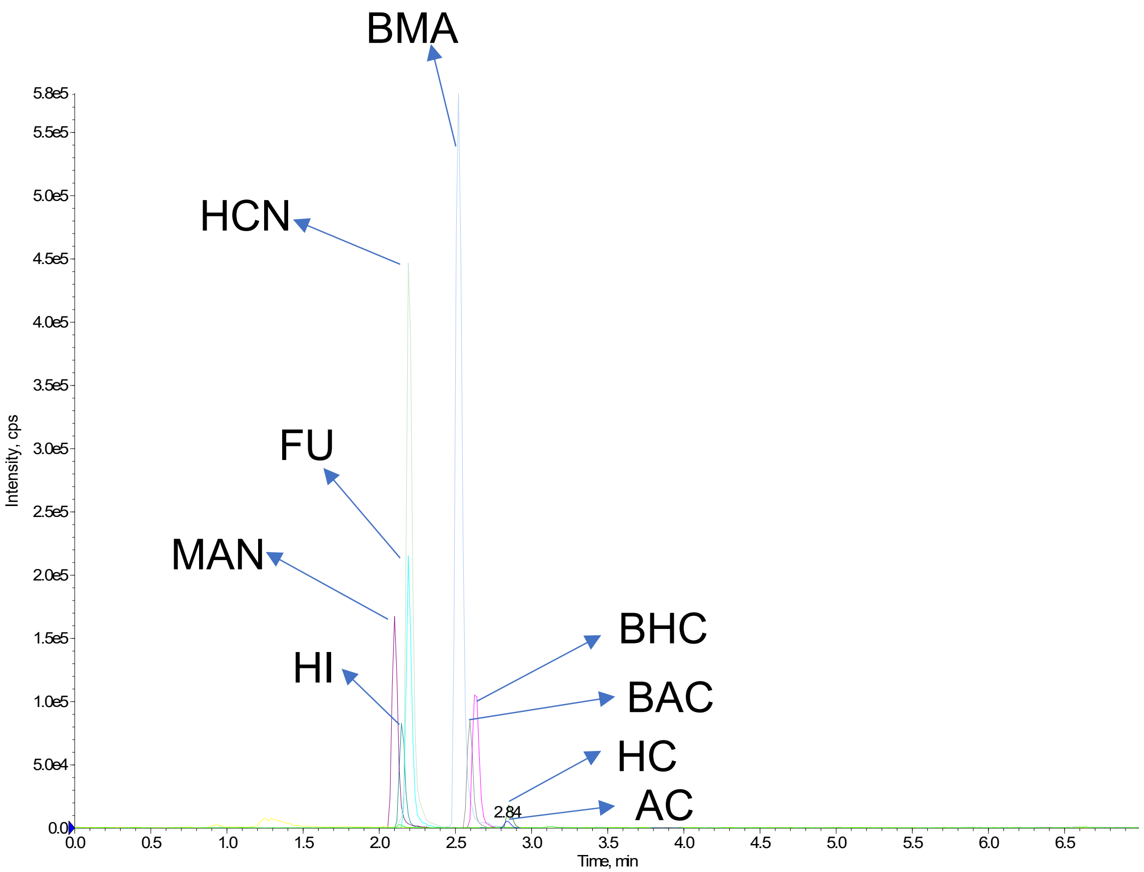

UPLC-MS/MS methods:

Reference solution preparation:Weigh accurately aconitine (AC), mesaconitine (MA), hypaconitine (HC, benzoylaconine (BAC), benzoylmesaconine (BMA), benzoylhypaconine (BHC),conine (ACN), mesaconine (MAN), hypaconine (HCN), salsolinol (SA), fuziline (U), higenamine (HI) in suitable amount, put in a 100 mL brown volumetric flask, and dissolve and dilute to the with methanol to make the mixed reference solution mother liquor with the quality concentration of AC, MA, HC, BAC, BMA, BHC, ACN, MAN,CN, SA, FU, HI as 0.001 03, 0.001 02, 0.002 30, 0.000 83, 0.010 24, 0.000 7, 0.00 88, 0.011 5, 0.001 84, 0.000 17, 0.02 62, 0.000 010 2 mg/mL, respectively.

Sample preparation method: Take about 2 g of UAP, SFAP, GCAP, and DGSAP powders (passed through a No.3 sieve) separately, weigh accurately, put them in a stoppered conical flask, add 3 mL of ammonia test solution, add 50 m of isopropanol-ethyl acetate (1∶1) mixed solution precisely, weigh the mass, ultrasonic treatment for 30 min, cool, use isropanol-ethyl acetate (1∶1) mixed solution to make up for the loss of mass, shake well, and filter. Accurately measure 25 mL the filtrate, recover the solvent to dryness under reduced pressure at below 40 ℃, add 25 mL of methanol solution to the residue precisely dissolve it, take 1 mL of the methanol solution, add water to 5 mL, filter, and take the filtrate, each extract of the herbal, each sample is extracted 3 times, and the content is averaged.

Chromatographic conditions: ACQUITYPLC BEH C18 chromatographic column (2.1 mm × 100 mm, 1.7 μm); mobile phase of 0.1 formic acid aqueous solution (A) - acetonitrile (B); gradient elution (0–2 min, 10%–70%; 2–4 min, 70% B; 4–6 min, 70%–90% B; 6–6.1 min 90%–5% B; 6.1–7.0 min, 5% B). Flow rate 0.3 mL·min-1 sample injection volume 1 μL; column temperature 20 ℃.

MS conditions: ion source was Turbo Spray; multiple reaction monitoring (MRM) mode; collision gas was nitrogen; curtain gas pressure (CUR) 1 psi (1 psi ≈ 6.895 kPa); collision gas pressure (CAD) 9 psi; spray voltage (IS) 450 V; nebulizing temperature (TEM) 550 ℃; nebulizing gas (GS1) 55 psi; auxiliary gas pressure (GS2) 50 psi.

Thin layer chromatography analysis

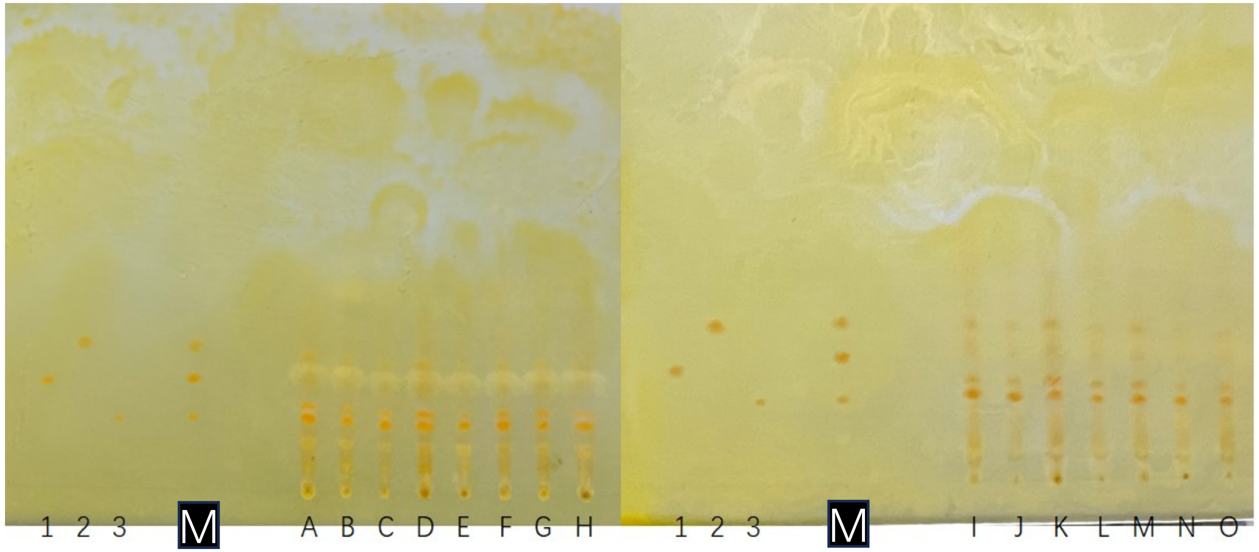

1： Benzoylaconine； 2： Benzoylmesaconine； 3： Benzoylhypaconine； M 1, 2, 3 mixed standard;
A-O： Different batches of SFAP

Preparation of sample solution:

Take 4 g of the powder of this product, first moisten it with 6 ml of ammonia test solution, then add 50 ml of ether, fully oscillate and extract it with an ultrasonic device for 30 minutes. After the extraction is completed, separate the solid liquid by filtration operation, collect the filtrate and evaporate it completely. Next, the obtained dry residue is dissolved with 0.5 ml of dichloromethane prepare the sample solution.

Preparation of standard solution:

Take the standard in BAC, BMA, BHC, and mix it with isopanol-dichloromethane (1:1) to prepare a mixed solution containing 1 mg per 1 ml as the standard solution.

Preparation of negative control solution:

Prepare isopropanol-dichloromethane (1:1) mixed solution as negative control solution

Fingerprint pattern analysis

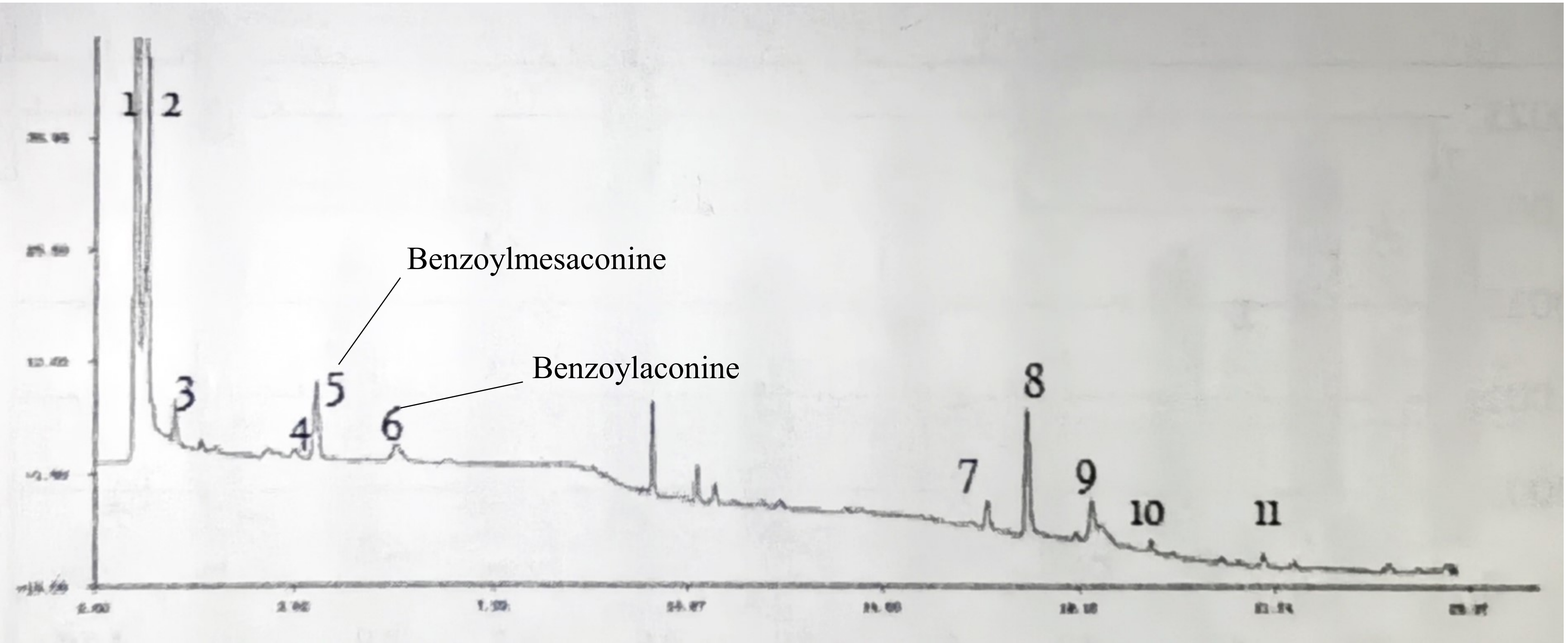

Two characteristic components were identified as 5、6

Fingerprint pattern methods:

Chromatographic conditions: Waters BEHC18 chromatographic column (2.1x50mm, 1.7μm), phase: acetonitrile (A) - 40 mmol L ammonium acetate (adjusted to pH = 10 with ammonia water), elution procedure was 0-7 min, 26%-30% A; 7-8 min. 30%-47% A; 8-3 min. 47%-51% A: 13-20 min. 51%-80% A; 20-25 min 80% A. Flow rate 0.3 ml min, column temperature 30 ℃, detection wavelength 235 nm, sample injection volume 2μL.

Preparation of standard solution: A certain amount of benzoylmesaconine and benzoyl aconine was accurately weighed and put into a 5 millimetric flask, and then dissolved in methanol to make a mixed standard solution with a concentration of 0.01785 mg/ml and 0.09 mg/ml.

Preparation of sample solution: Accurately weigh 1 g of powder and put it into a conical flask with a stopper, and methanol precisely. 30ml, ultrasonic extraction for 50min, filtration, the filtrate was concentrated to dryness, the residue was dissolved in 005% hydrochloric acid-methanol, transferred to a 5ml volumetric flask, diluted to the mark, mixed well, and the solution was.
